# Ion-Imprinted Chitosan Technology for Heavy Metal Ion Removal from Water and Wastewater: A Review on Recent Insights and Future Perspectives

**DOI:** 10.3390/ijms27073183

**Published:** 2026-03-31

**Authors:** Łukasz Wujcicki, Joanna Kluczka

**Affiliations:** Department of Inorganic, Analytical Chemistry and Electrochemistry, Faculty of Chemistry, Silesian University of Technology, B. Krzywoustego 6, 44-100 Gliwice, Poland

**Keywords:** ion-imprinting, biopolymer, chitosan, IIP, metal ions, selective, adsorption, water treatment, wastewater

## Abstract

Ion-imprinting technology based on biosorbents via sorption demonstrates potential for the selective removal of metal ions from water and wastewater. This offers both high sorption capacity and selectivity for specific metals. Current research trends are toward the development of sorbents with minimal environmental impact. Among the most rapidly evolving classes of sorbents are those derived from biopolymers, such as chitosan—a natural derivative of chitin that can be readily functionalized. Due to the growing interest in this topic, it is necessary to summarize the current knowledge. In this article, we provide a comprehensive overview of the latest advances in ion-imprinted chitosan-based materials designed for the purification of metal-contaminated aqueous systems. We conduct a bibliographic analysis and describe a variety of chitosan-based materials exhibiting selectivity toward heavy metals, including chromium Cr(III/VI), cobalt Co(II), nickel Ni(II), copper Cu(II), zinc Zn(II), arsenic As(III/V), cadmium Cd(II), mercury Hg(II), and lead Pb(II). Finally, we discuss future prospects and highlight current research gaps, aiming to guide further scientific exploration and innovation in this promising field.

## 1. Introduction

Heavy metals, including chromium, cobalt, nickel, copper, zinc, arsenic, cadmium, mercury, and lead, cause significant environmental problems due to their toxicity, persistence, and tendency to accumulate in ecosystems [1,2,3,4,5,6]. The primary sources of water pollution by these metals are the release of untreated or insufficiently treated wastewater from domestic, industrial, and agricultural sectors [7,8,9]. Such contamination degrades aquatic ecosystems, facilitates the spread of waterborne diseases, and diminishes the availability of clean water resources [10]. Addressing these challenges, the removal of metals from water and wastewater not only mitigates environmental risks but also enables the recovery of valuable raw materials, such as cobalt, copper, and nickel, which are essential for the economy of the European Union (EU). This connection is emphasized by the European Union Directive 2024/1252 of 11 April 2024, which underscores the necessity of securing raw material supplies for the EU economy and internal market, particularly for critical metals [11]. Thus, addressing the dual objectives of water purification and critical metal recovery necessitates comprehensive treatment strategies. In response, researchers are developing advanced technologies to improve the effectiveness and efficiency of wastewater purification processes [12]. These technologies include chemical precipitation [13,14], extraction [15,16,17], adsorption [18,19], flocculation [20], membrane filtration [21], and ion exchange [22,23]. Building on these technological advancements, recent efforts have also focused on material innovations for purification, particularly the incorporation of new polymers and techniques.

Ion-imprinting technology has recently attracted significant attention as a promising approach for selectively removing heavy and critical metals from water and wastewater [24]. Building on previous innovations in purification, this technique involves creating specific binding sites within a polymer matrix. These sites are tailored to the size and “shape” of a particular charged particle [25]. By incorporating these ion-specific binding sites, the resulting sorbent can efficiently capture target metal ions, even in the presence of other competing ions [26]. The preparation of ion-imprinted sorbents typically begins with the complexation of the target ion (serving as a template) with functional monomers or polymers. The resulting complex is then crosslinked to form a stable polymeric matrix that permanently incorporates the imprinted binding sites. Following the removal of the template ions, the material retains well-defined cavities complementary to the target ions, resulting in high selectivity for their subsequent binding.

In recent decades, interest in the application of natural materials, such as biopolymers (e.g., alginate, pectin, chitin, and chitosan) has grown. The latter material is considered one of the most promising biopolymers for the development of advanced materials due to its favorable properties. These include abundance, nontoxicity, biodegradability, and biocompatibility [27]. In the case of chitosan, the hydroxyl and amino groups in its structure can coordinate with metal ions to form stable chelates [28]. However, chitosan’s poor physical properties and high degree of swelling in aqueous systems limit its practical application. At low pH, protonation of the amino groups in chitosan and its composites enhances adsorption via electrostatic interactions and chelation. Under these conditions, hydrolytic degradation of chitosan glycosidic linkages may occur, weakening the polymer backbone, promoting dissolution, and ultimately reducing mechanical and structural stability [29]. Therefore, research has increasingly focused on strategies to strengthen the physicochemical stability of chitosan matrices and to optimize their selective removal performance for specific contaminants.

**Figure 1:**
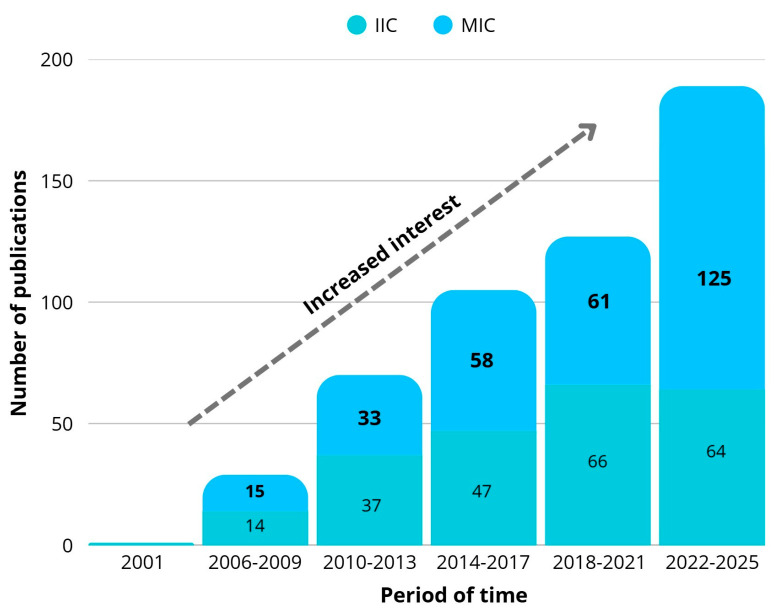
Number of publications related to the use of chitosan in molecular- and ion-imprinted technology as composites in 2001–2025 using the SCOPUS database (last access on 24 January 2026). IICs—chitosan AND ion-imprinted OR “IIP” OR “IIPs”), MICs—(chitosan AND molecular-imprinted OR “MIP” OR “MIPs”). Source: Own search.

The first ion-imprinted polymers (IIPs) were described by Nishide et al. in 1976 [30]. In their study, they introduced a novel resin prepared by crosslinking 4-vinylpyridine (4-VP) with 1,4-dibromobutane in the presence of different metal ions as templates. Nevertheless, the real development of IIPs is more recent, and a considerable increase in publications on IIPs has occurred over the last 20 years. In the next section, we discuss the bibliometric analysis of these trends. A similar trend can be observed for molecular chitosan (MIC), as illustrated below (Figure 1).

**Figure 2:**
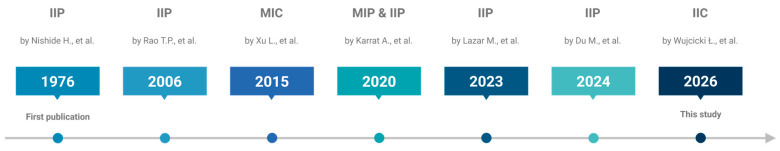
Evolution of review articles about IIPs and MIPs [30,31,32,33,34,35]. Source: Own search.

In the current century, a few review articles have been dedicated to IIPs. Notable examples are those from 2006, 2023, and 2024 published by Rao T.P., Lazar M.M., and Du M., respectively [31,32,33]. They mainly focused on the principles and applications of different types of IIPs. Also, for the application of chitosan, two review articles can be noted: one from 2015 and the other from 2020, by Xu L. and Karrat A., respectively [34,35]. However, those articles focused more on molecular rather than ion-imprinting (focusing on the removal of neutral molecules rather than charged metal ions) (Figure 2).

Despite their potential, molecular or ion-imprinted polymers are still rarely used at an industrial scale due to their limited efficiency. This drawback, however, could be addressed by using inexpensive, renewable, and environmentally friendly polymers. To the best of our knowledge, no previous review has comprehensively examined the application of chitosan in ion-imprinting for metal-ion removal, making this review a novel contribution to the field. Existing reviews either focus broadly on molecular or ion-imprinting techniques, and discuss chitosan-based sorbents without specifically considering the imprinting approach. Therefore, this review offers a unique and focused perspective by integrating these two areas.

The novelty of this work, therefore, aims to fill this gap by focusing on IIC’s for metal ion recognition, with particular emphasis on studies published since 2000. The added value of this review is to provide chemists with guidance for designing IIC sorbents, including general principles, characterization methods, design strategies, and the different material formats available for ion-imprinting.

The main part of the article is divided into subsections, each focusing on a specific metal. These subsections discuss the most important information about sorbents used for the selective removal of a given metal. They cover, among other things, sorbent synthesis methods, their sorption capacity and selectivity, and other interesting aspects that, in the authors’ opinion, deserve attention. For consistency, the broader term “sorption” is used throughout this review when referring to adsorption, regardless of article type.

## 2. Bibliometric Study

Bibliometric analyses offer a comprehensive quantitative framework for evaluating scientific productivity, revealing research trends, thematic evolution, and structural indicators like publication growth, leading countries, collaboration networks, and dominant keywords [36]. Building on this, a dedicated bibliometric search was conducted to assess studies focused on ion-imprinted chitosan materials for the selective removal of heavy metal ions from aqueous solutions.

### 2.1. The Search Strategy

Searches were performed in the Scopus database using structured queries with field-specific tags. The TITLE-ABS-KEY field was used to ensure comprehensive coverage of relevant records. Search strings captured studies on chitosan-based ion-imprinted polymers and their sorption applications. For example: TITLE-ABS-KEY (“chitosan”) AND TITLE-ABS-KEY (“ion-imprinted” OR “IIP” OR “IIPs”) AND TITLE-ABS-KEY (“adsorption” OR “sorption”) AND TITLE-ABS-KEY (“chromium”) AND TITLE-ABS-KEY (“selective removal”). Similar queries were created for other target heavy metals. All searches used English keywords. No initial restrictions on publication language, document type, or full-text access were applied to ensure maximum inclusivity during identification.

The search covered publications from 2003 to the end of 2025 available in the Scopus database. All retrieved records were exported in BibTeX format. Each record included information on authors, title, source, year of publication, DOI, abstract, and keywords. The datasets were subsequently imported, cleaned, and preprocessed in RStudio 4.5.3 and the Bibliometrix package to ensure reproducible bibliometric analysis [37].

### 2.2. Study Selection Process

The study selection process commenced following the finalization of the search strategy. In total, 237 records related to ion-imprinted chitosan were identified, of which 68 specifically addressed heavy metal ion removal (chromium: 5; cobalt: 4; nickel: 11; copper: 18; zinc: 2; arsenic: 5; cadmium: 8; mercury: 2; lead: 13). All raw datasets (BibTeX files) and R scripts are provided in the Supplementary Materials to ensure transparency and reproducibility. Visualization of bibliometric networks was also performed using the VOSviewer software 1.6.20 to enhance the interpretability and clarity of the results.

**Figure 3:**
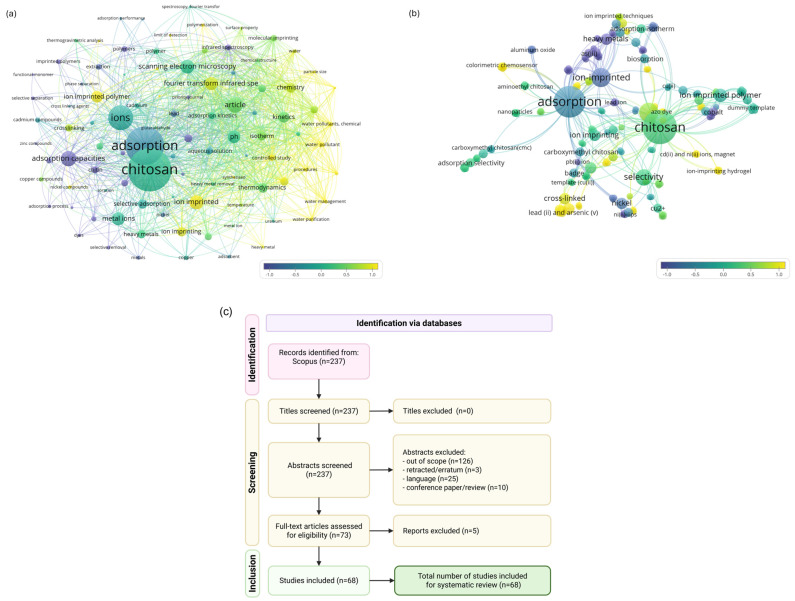
Keyword co-occurrence networks on chitosan-based ion-imprinting research before (**a**) and after screening (**b**) according to the PRISMA flow diagram (**c**). Source: Own search.

The study selection process followed a PRISMA flow diagram, including title screening, abstract screening, and full-text assessment (Figure 3c). At the screening stage, all 237 records were evaluated based on titles and abstracts.

A total of 164 records were excluded after abstract screening based on predefined exclusion criteria. The main reasons for exclusion were studies outside the scope of this review (n = 126). These included publications that mentioned chitosan, ion imprinting, or heavy metals but did not describe the adsorption synthesis or application of ion-imprinted materials in water treatment. Other exclusions were retracted papers or errata (n = 3), articles in languages other than English (n = 25), and document types such as conference papers and review articles (n = 10).

After this stage, 73 full-text articles were assessed for eligibility. During full-text evaluation, 5 more studies were excluded manually. These were removed due to insufficient methodological relevance or insufficient experimental detail regarding material synthesis or sorption performance.

Ultimately, 68 studies met all inclusion criteria and were included in the final qualitative and bibliometric analysis.

### 2.3. Bibliometric Analysis

Two separate analyses were performed. The first included all publications on ion-imprinting in chitosan materials. The second focused on selected studies on the applications of chitosan-based ion-imprinted materials for the selective removal of heavy metal ions. These ions included chromium Cr(III/VI), cobalt Co(II), nickel Ni(II), copper Cu(II), zinc Zn(II), arsenic As(III/V), cadmium Cd(II), mercury Hg(II), and lead Pb(II).

Keyword analysis revealed the main research directions within the field. The term “chitosan” appeared most frequently (173 occurrences), followed by “adsorption” (154 occurrences), “ions” (99 occurrences), “adsorption capacities” (61 occurrences), and “ion-imprinted” (51 occurrences). These findings indicate that research efforts are primarily focused on chitosan-based sorption processes, which dominate the current bibliometric landscape. At the same time, the increasing use of terms related to ion-imprinting suggests that this area is an emerging, rapidly developing research trend, as illustrated in Figure 3a.

A similar pattern was observed in the second analysis, which specifically addressed studies concerning heavy metal removal (Figure 3b). Here, the research trend, marked in yellow and green, emphasizes improved selectivity, material crosslinking strategies, and the application of ion-imprinting techniques to enhance sorption performance. Overall, the results demonstrate not only the continuous growth of this research field but also the need for sustained investment in further studies and innovation. In particular, interdisciplinary approaches aimed at developing practical, scalable solutions to environmental challenges related to ion-imprinting technologies are particularly important.

Apart from the keyword analysis, attention should also be paid to the authors’ countries of affiliation in the examined publications (see Supplementary Materials). The majority of contributions originated from China (89 occurrences), followed by Saudi Arabia (31), Egypt (27), and even Poland (11). This geographical distribution suggests not only a concentration of expertise in specific regions but also potential differences in research priorities and application domains. The strong dominance of Chinese institutions may be linked to intensive governmental funding, rapid technological development, and a high publication output in engineering and applied sciences. At the same time, the limited representation of other regions indicates that the method has not yet achieved widespread acceptance. As awareness grows and successful case studies accumulate, the research field is likely to transition from a niche topic toward more mainstream scientific and industrial applications.

## 3. Chitosan—Base Material for Ion-Imprinting Process

The range of publications that discussed the properties of chitosan is extensive and worth recommending [38,39,40,41,42]. We intend to present new and interesting facts about chitosan in terms of economics, sources of chitosan, and changes in its occurrence, among others. The growing interest in chitosan and its modifications has led to a steady increase in published articles. As of 2024, there have been over 12,000 articles on this subject in the Scopus database. A bibliometric analysis of chitosan as a biopolymer was also conducted by Muñoz F.L. et al., focusing on its industrial applications [43]. One of the key conclusions of this study is the growing interest in chitosan, particularly in the agricultural, pharmaceutical, and water treatment industries.

### 3.1. Chitosan Principles and New Insights

Chitosan is widely recognized as the most important derivative of chitin, from which it is produced through a deacetylation process [44]. Both biopolymers belong to the group of linear polysaccharides. Chitosan is composed of a random arrangement of D-glucosamine residues linked by β-(1,4) bonds and N-acetyl-D-glucosamine. Chitosan is readily available due to the widespread occurrence of its precursor, chitin, which can be extracted from crustacean exoskeletons (shrimp, prawns, crabs, and lobsters), insect shells, algae, and fungal cell walls [45]. By source, the largest revenue is generated from shrimp chitosan production (63% in 2023) [46]. However, there has also been an increase in the extraction of chitosan from fungal sources [47].

Its properties, including biocompatibility, biodegradability, and antimicrobial activity, make it a versatile material with applications in various fields, such as medicine, agriculture, water treatment, the food industry, and even winemaking. We can already see new reports of chitosan use across various areas of life. One interesting example is Dyson’s use of chitosan, which introduced its first haircare and styling products in 2024 [48]. Chitosan can also be used as a water-soluble coating that extends the shelf life of raw eggs. This coating preserves egg quality and prevents bacterial contamination, keeping eggs edible for up to seven weeks at room temperature, compared to the usual two to four weeks for uncoated eggs [49].

The primary distinction between chitin and chitosan lies in their solubility and degree of deacetylation. When the degree of deacetylation in chitin exceeds 50%, it is referred to as chitosan. This transformation improves the material’s properties, primarily by reducing the content of acetamido groups. As a polycationic polymer, chitosan contains a positively charged amino group (pKa = 6.5), which enhances its solubility in acidic and neutral solutions. The properties of chitosan are influenced by factors, namely its molecular weight, degree of acetylation, solution pH, temperature, polymer crystallinity, and its physical and morphological form (powder, granulate, flake, membranes and films, fibers, hydrogels, nanoparticles, and microspheres) [50]. Its solubility characteristics can be modified by altering its molecular weight or degree of deacetylation. Furthermore, the presence of functional hydroxyl (-OH) and amino (-NH_2_) groups enables easy modification via grafting and crosslinking [51]. The primary amine groups in chitosan allow for various chemical modifications, enhancing its functionality and enabling it to interact with other molecules [52]. This property is exploited in applications such as heavy metal ion removal from wastewater and drug encapsulation. Chitosan’s structure closely resembles that of cellulose, with the key difference being the amino group at the C-2 position instead of a hydroxyl group. This structural variation significantly broadens the potential applications of chitosan.

Furthermore, the U.S. Food and Drug Administration has recognized chitosan as a safe compound, which supports its potential applications in various sectors, particularly in biomedicine and agriculture [53].

### 3.2. Economic Aspects of the Chitosan Market

**Figure 4:**
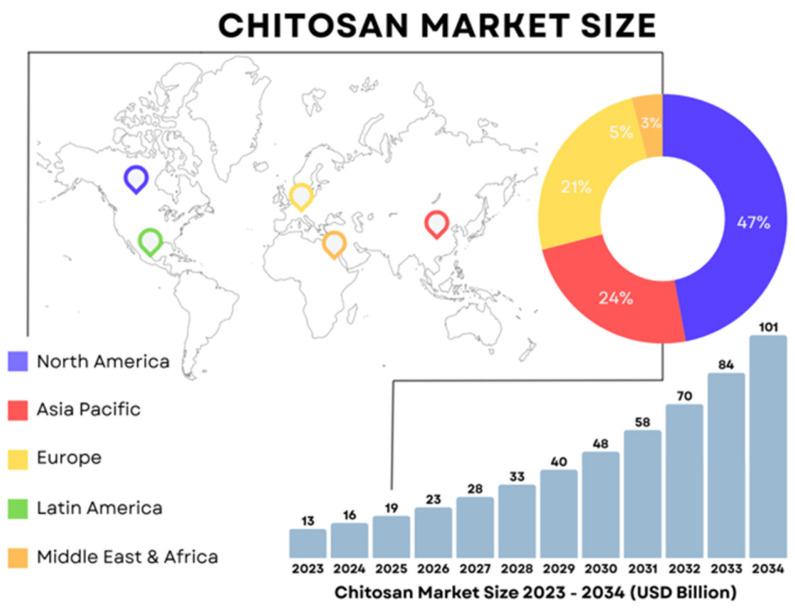
Chitosan market size 2023–2034 [54].

The chitosan market has gained attention for its diverse applications and strong economic prospects. In 2024, the global market size was estimated at $15.92 billion, growing to $19.15 billion in 2025, and is projected to reach approximately $101 billion by 2034, with a CAGR of 20.29% (Figure 4) [54]. Compared with estimates from the mid-2010s, the global market has already multiplied several dozen times, confirming remarkable progress in large-scale chitosan production (from $0.48 to $15.92 billion from 2016 to 2024) [55]. North America currently represents one of the major centers of chitosan utilization, at 47% of the market, supported by favorable regulatory frameworks and a well-established manufacturing base. This dynamic growth underscores the strategic importance of chitosan, not only in biomedical, agricultural, and food-related fields, but also as a sustainable material for environmental applications, particularly wastewater treatment and metal ion removal.

All indications suggest that the Asia Pacific region will capture a larger share of chitosan production between 2025 and 2034. This growth is driven by the rapid expansion of the fish industry in these countries, particularly shrimp production, which currently leads in chitosan production due to favorable extraction conditions (e.g., a high chitin content in the form of alpha-chitin). In 2024, China produced 3.7 million tons of shrimp. The IMARC Group estimates that the market will reach 5.1 million tons annually by 2033, exhibiting a CAGR of 3.65% from 2025–2033 [56].

Shrimp shells remain the primary industrial source of chitosan, as they contain 15–40% chitin, while crab shells contain 15–20% [57]. Squid also possesses a relatively high chitin content (35–50%), but their limited availability, variable composition, and higher processing costs make shrimp the dominant raw material for large-scale production (Table 1). India, as one of the largest global producers and exporters of shrimp, plays a particularly important role in sustaining chitosan supply [58].

### 3.3. Chitosan Main Source and Costs

**Figure 5:**
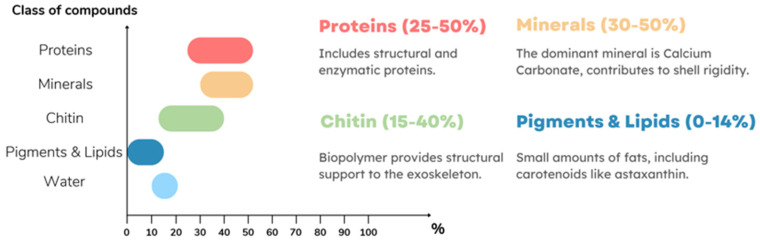
Average starting composition of shrimp shell [59,60].

The largest current source of chitosan is the exoskeletons of marine animals, predominantly shrimps, as mentioned earlier. Shrimp shells are made up of densely woven matrices of chitin fibers intertwined with proteins. These matrices are reinforced by the deposition of mineral salts, primarily calcium salts. Additionally, pigments and lipid compounds are present in the shells, with their quantities and properties varying between species and individuals. These variations depend on factors including growth stage, sex, diet, and environmental conditions. Different biological species exhibit structural differences, resulting in a broad range of chitin, proteins, minerals, and pigments in their shells (Figure 5). Typically, shrimp exoskeletons contain approximately 25–50% protein, 30–50% minerals (mainly calcium carbonate), 15–40% chitin, and 0–14% other compounds, such as pigments (e.g., astaxanthin) and lipids (including muscle residues and carotenoids) [59,60].

The costs of chitosan depend on various factors, including purity, molecular weight, degree of deacetylation (DDA), and application. Additionally, seasonal seafood production, environmental policies, and supply chain disruptions also influence its pricing. The global price of chitosan fluctuates due to raw material availability, processing costs, market demand, and international trade regulations. As a result, the chitosan that is used in industrial applications (e.g., water treatment and agriculture) as bulk chitosan is more affordable, ranging from $10 to $50 per kg, while high-purity chitosan for medical or pharmaceutical use can cost between $100 and $1000 per kg (Table 2).

In conclusion, chitosan’s unique properties and versatile applications make it a material of considerable interest in both the industrial and scientific fields. Innovations in production technologies and an increasing focus on sustainability are likely to enhance its market potential, positioning chitosan as a key player in green and biotechnological advances in the decades to come.

## 4. Selective Sorption in Ion-Imprinting Technology

### 4.1. Selectivity in the Sorption Process

Selectivity refers to the ability of a material or chemical compound to specifically adsorb or absorb certain molecules or ions compared to others [62]. This property is crucial across various fields, such as chemistry, environmental science, and medicine. In sorption, selectivity is primarily determined by specific interactions between the sorbent and the sorbate. These interactions can include chemical bonding, van der Waals forces, hydrogen bonding, and other molecular forces [63].

The selectivity of a sorbent is influenced by its chemical composition, structure, and surface properties [63]. In separation processes, such as chromatography, sorption selectivity is vital for achieving the desired separation of components in a mixture. Selective sorption refers to the ability of a material to exhibit a strong affinity toward a specific sorbate. At the same time, it minimizes interactions with other components in a mixture, enabling the targeted removal or separation of that substance [64]. Several approaches can be employed to achieve selective sorption. One of the most important steps is selecting a suitable sorption material that exhibits a high affinity for the target molecule. This material should possess a defined structure and composition, along with functional groups that contribute to its selective removal capabilities [65]. If the chosen material lacks selectivity, adjusting sorption parameters, such as solution pH, may be necessary. Key parameters include the solution’s reaction conditions, temperature, and the contact time of the material with the mixture containing the target molecule and competing substances. If a particular material lacks high selectivity, modifications to its surface properties may be needed. This can involve functionalizing or doping the material to enhance its selectivity. Increasing the surface area of the modified material can also generally boost its sorption capacity [66].

The introduction of other compounds can significantly affect the sorption parameters and may even alter the sorption mechanism entirely. Recently, computational methods have been employed to expedite the design of sorbents capable of binding specific types of molecules [67,68]. Selecting material that exhibits selectivity without modification is often challenging, which leads to further research and implementation of modifications. Such modifications typically relate to the addition, removal, or replacement of functional groups on the material’s surface.

In the context of metal ion sorption, there is a trend toward incorporating organic molecules (often polymeric) into the sorbent structure. These molecules possess complex-forming properties related to the targeted metal. A notable example is the ion-imprinting technique, which creates specific sites within a polymer structure capable of binding metal ions with distinct properties [69]. Bhaskarapillai A. et al. conducted studies that mathematically described selective sorption, focusing on an IIP and examining complexation reactions between the metal ions Co(II), Cu(II), Fe(II), and Ni(II) and N-(4-vinylbenzyl)iminodiacetate [70]. Their research demonstrated that even when the ionic radii and charge-to-radius ratios of various metal ions are nearly identical, interactions within the matrix can exhibit distinct energy densities, leading to selective recognition of the template ion.

To assess the effect of selectivity in imprinting techniques, comparisons need to be made between the sorption properties of the imprinted material and those of a non-imprinted material, referred to as the imprinted polymer (IP) and the non-imprinted polymer (NIP) (or controlled polymer (CP)), respectively. Both types of sorbents should undergo analogous testing procedures, which involve sorption in a solution containing the target metal ion and in mixtures containing the target metal ion alongside competing ions. This can be carried out through two methods: batch sorption or flow sorption (column sorption) [71].

### 4.2. Mathematical Considerations in Selective Sorption Processes

Based on the analysis of the ion concentrations in solution after contact with the material, two important values can be determined: recovery percentage/removal percentage (*R*%) and sorption capacity (q), calculated using the following equations [72]:

(1) $$R\%=\frac{C_{0}-C_{e}}{C_{0}}\cdot 100\;[\%]$$

(2) $$q=\frac{(C_{0}-C_{e})\cdot V}{m}\;[\frac{mg}{g}]$$

where *C*_0_ and *C_e_* are the concentrations of the metal in solution before and after sorption (at equilibrium) [mg/dm^3^], *V* is the volume of the solution [dm^3^], and *m* is the mass of the sorbent used in the process [g].

However, these formulas may not fully characterize the efficiency of selective sorption. The imprinting phenomenon refers to the ability of an ion template to be recognized by an IIP sorbent in the presence of other ions [73]. To study this selectivity, sorption is performed in a solution containing both the template ion and competing ions. Preliminary calculations focus on determining the partition constant (*K_d_*), which represents the ratio of sorbate (the template ion or competing ion in this case) partitioning between the solid phase (sorbent) and the liquid phase (solution) at equilibrium. Next, the ratio of the *K_d_* of the template ion to that of the competing ion, known as *K_IP_*_/*NIP*_, is calculated. The final step involves calculating the relative selectivity coefficient (*k*′), which indicates the ratio of the ion partition coefficients in the IP compared to the NIP [74]. A higher value of the constant *k*′ indicates a stronger imprinting effect. Mathematically, this process can be described by the following formula:

(3) $$K_{d}\left(ion\;template\;or\;competitive\;ions\right)=\frac{q_{e}}{C_{e}}\;[\frac{dm^{3}}{g}]$$

(4) $$k_{IP/NIP}=\frac{K_{d}(ion\;template)}{K_{d}(competitive\;ion)}$$

(5) $$k^{\prime }=\frac{k_{IP}}{k_{NIP}}$$

where *q_e_* and *C_e_* represent the sorption capacity and concentration, respectively, of the template/competitive ion in solution at equilibrium.

When characterizing the sorption process using isothermal and kinetic methods, it is essential to conduct tests on both the NIP and IP. Based on the authors’ experience, the concentration of metal ions in solution significantly impacts selectivity. Therefore, it is advisable to investigate and report on the concentration range that yields the highest selectivity coefficients.

## 5. Classification of the Techniques Used in Ion-Imprinting Technology

Currently, imprinting technology is an important area within the field of sorption processes, particularly concerning the utilization of molecularly imprinted polymers (MIPs) and IIPs. Recent advances in the synthesis of materials based on these polymers have been significant, with applications in environmental, food, and pharmaceutical analysis, among others. This technology is especially effective for the selective recognition of metal ions, with studies demonstrating improved recognition rates for Cu(II), Ni(II), and Zn(II) ions [75]. Surface-imprinted ionic materials provide specificity and stability for the sorption of various heavy metals [76]. However, it is important to note that IIPs remain in the early stages of development, and further research is needed to fully realize their potential [77,78].

### 5.1. Chemical Immobilization

Chemical immobilization works similarly to the synthesis of molecularly imprinted polymers, involving a ligand molecule with or without a polymerizable functional group. When a ligand, a target metal ion, and a crosslinking agent are mixed, polymerization creates a matrix with active sites. After eluting the metal ion, the specific active site remains. This process often entails forming metal ion complexes with ligands containing vinyl groups, followed by polymerization using monomers. Researchers commonly synthesize these in a single step by combining the metal ion, monomer, and crosslinking agent prior to copolymerization. Various organic compounds have been studied, with initial monomers such as 4-VP having been used to immobilize copper(II) and mercury(II) ions for sensors [79,80]. However, such monomers may show low selectivity, prompting efforts to enhance ligand properties for better recognition. As a result, researchers functionalize complex ligands for polymer networks. Ligand–metal interaction can occur in two ways:

(1) Single-step process: Here, the ligand–metal complex is directly copolymerized, simplifying the synthesis. For instance, Giovea A. et al. produced a Ni(II) IIP using 2-(aminomethyl)pyridine as the ligand [81].

(2) Two-step process: This method involves first forming and isolating the ligand–metal complex before copolymerization, allowing greater control over the final product. One notable study detailed the preparation of a Co(II) ion-imprinted sorbent for reducing radioactive waste, using N-(4-vinylbenzyl)iminodiacetic acid and copolymerizing the Co(II) complex with ethylene glycol dimethacrylate and 2-azobisisobutyronitrile (AIBN), achieving high selectivity for metal ions such as Fe(II) and Ni(II) [82].

### 5.2. Trapping

The chemical immobilization technique previously discussed uses specific monomers to extract targeted metal ions through ion-imprinting. Since these monomers are not commercially available, they must be synthesized in the lab, which requires significant time and financial resources. Additionally, modifying the resulting complex ligands, for example, by adding vinyl groups, can be difficult.

To address these challenges, researchers are increasingly using ligands without functional groups capable of polymerization. Instead of forming permanent interactions with the polymer matrix, these ligands are incorporated through mechanical retention, saving time. This approach was first introduced in 2003 by Biju V.M. et al. for the separation of dysprosium(III) [83]. In this process, a complex of Dy(III), 5-7-dichloroquinolinol, and 4-VP was formed, where the ligand was trapped within the polymer, allowing for selective Dy(III) ion removal, despite the presence of Y(III) ions. Since then, the trapping method has gained popularity for “imprinting” various metal ions with different ligands. For instance, Metil P. et al. showed that simple, low-cost ligands could be used to create resins with high sorption capacity and selectivity for U(VI) ions [84]. Despite this method’s advantages, challenges remain. It is crucial to ensure that the ligand is properly incorporated into the polymeric network and does not leach during ion removal. There are two routes for synthesizing polymers using the trapping technique: preparing the complex in situ before polymerization or isolating it beforehand.

The main advantage of the trapping technique is its simplicity, which allows researchers to avoid the tedious preparation of vinylated ligands. However, some prefer to form chemical bonds for added stability and reusability. Ultimately, the choice between chemical immobilization and trapping may depend on the higher costs associated with synthesizing vinylated ligands.

### 5.3. Polymer Chain Cross-Linking

The crosslinking of linear polymer chains that have functional groups capable of binding metal is one of the oldest techniques used in the IIP preparation procedure. In 1976, as above-mentioned, Nishide et al. copolymerized 4-VP with 1,4-dibromobutane in the presence of Cu(II), Fe(III), Co(II), Zn(II), Ni(II), or Hg(II) [85]. However, this procedure is most commonly used for selective cation removal using natural linear polymers, i.e., cellulose and chitosan. Hashem A. et al. used an extracted pulp from sunflower stalks containing 99.5% alpha-cellulose [86]. This cellulose was then grafted with acrylamide, and the resulting material was used to remove Hg(II) ions from aqueous solutions, a maximum sorption capacity of 625 mg/g being achieved with this sorbent. Chitosan, among natural polymers or polymers of natural origin, is distinguished by the presence of an amino group, which has a chelating character. Imprinting technology can help improve its stability through crosslinking. With chitosan, no radical polymerization is carried out, but it is crosslinked with agents such as epichlorohydrin (ECH), glutaraldehyde (GLA), sodium tripolyphosphate (STPP), genipin, citric acid, or sulfuric acid.

Chen et al. prepared chitosan imprinted microparticles using multiple metal ions, including Cu(II), Zn(II), Ni(II), or Pb(II), using ECH and GLA [87,88]. In their study, they assumed that the metal ions were chelated to the two polymer chains via amine and hydroxyl groups before crosslinking. Nishad et al. reported similar work using Co(II) and ECH crosslinking [89].

### 5.4. Surface Imprinting

Chemical immobilization and trapping techniques primarily utilize traditional polymerization methods, such as bulk, solvent, or suspension polymerization. While these methods achieve high sorption selectivity, they often reduce the sorption capacity because binding sites within the rigid polymer network are less accessible. Limited mass flow can also slow sorbate removal, negatively affecting template extraction from the polymer matrix and decreasing sorption capacity as selectivity increases. To address these issues, the surface imprinting technique creates active sites on the polymer’s surface, ensuring the complete removal of metal ion templates, enhancing accessibility to target ions, and reducing mass-transfer resistance.

A common method for achieving surface imprinting is emulsion polymerization, in which an amphiphilic ligand forms a complex with a metal ion template at the emulsion interface. The resulting cavities are mainly located on the material’s surface.

Recent advances in surface imprinting have focused on modifying small-sized particles to create core–shell materials that improve the efficiency of chromatographic columns [90]. Typically, inorganic particles, such as silica, are used for their stability and ease of functionalization. In some cases, biomass, such as waste mycelium, has also been used as a core material, coated with imprinted chitosan [91].

## 6. Mechanistic Study of Selective Binding Site Formation and Sorption Behavior Using Ion-Imprinting Technology

The concept of IIPs is similar to that of MIPs, but there are significant differences between the two techniques. While MIPs interact with template molecules through classical functional monomers, hydrogen bonds, or van der Waals forces, IIPs form interactions primarily through coordination bonds and electrostatic interactions [92]. Such interactions depend on the properties of the polymer matrix and even on the cross-linkers used [93]. In particular, the presence, type, and spatial distribution of functional groups (e.g., –NH_2_, –OH, –COOH) determine the strength and geometry of coordination interactions with metal ions, which directly influence both sorption capacity and selectivity [94]. Due to its interdisciplinary nature, achieving a full understanding of the ion-imprinting process is challenging, as it requires expertise from specialists across multiple fields.

In synthesizing IIPs, ligands or functional groups that can selectively bind to metal ions are predominantly employed [78]. These ligands are selected based on their donor atoms (N, O, S) and their affinity for specific metal ions, according to the hard–soft acid–base (HSAB) theory, which provides a physicochemical basis for selective sorption [95]. The general procedure for IIC synthesis involves forming a chitosan–metal or chitosan–ligand–metal complex, which is then copolymerized into the polymer network. The introduction of these ligands or complexes can occur through several methods, as above-mentioned, including the chemical immobilization of ligands, trapping unfunctionalized ligands within the polymer network, crosslinking linear polymer chains containing functional groups, and surface imprinting conducted in an aqueous-organic environment.

During ionic imprinting, the target metal ion serves as a template around which chitosan (or its derivatives) and cross-linking agents form a coating-like structure. In the initial stage, chitosan or its modifications form a complex with a given metal ion through electrostatic and coordination interactions, arising from the self-organization of chitosan’s functional groups. This self-organization is driven by the minimization of free energy, leading to an optimal spatial arrangement of functional groups around the metal ion that reflects its preferred coordination geometry (e.g., octahedral, tetrahedral) [96]. During cross-linking, the points of interaction with the matrix are fixed. The metal ion is then removed, most often by extraction with an acid solution or by using a ligand solution with a higher affinity for the metal, thereby forming cavities capable of selective rebinding [97]. The formation of these cavities can be demonstrated by comparing imprinted and non-imprinted materials using spectroscopic techniques, e.g., FTIR (shifts in peak positions can indicate the coordination of the metal ion template). Due to the surface’s geometric and chemical compatibility, these cavities preferentially bind template metal ions over other metals with different ionic radii and interaction strengths with specific functional groups [98]. As a result, a specific ‘ion memory’ is stored in the polymer, enabling selective re-binding of the target metal.

The binding sites formed are closely matched to the matrix in terms of the shape, size, and distribution of functional groups, but above all, energy [78]. This concept of “energy matching” means the correspondence between the binding energy of the cavity and the coordination energy of the metal ion template, which can be experimentally supported by adsorption isotherms (calculated K_L_), thermodynamic investigation (calculated ∆G), and even UV–Vis analysis (calculated degree of cleavage ∆ based on crystal field theory) [99].

The mechanism of heavy metal ion removal using ion-imprinting relies on the sorption of metals into active sites that, by definition, should be selective for a given metal ion. This mechanism primarily depends on the specific ion-imprinting method used. Regardless of the method, imprinting is associated with the formation of coordination compounds [32]. Heavy metals of the d-block form numerous complex compounds, and even in water, metal cations do not exist in their bare form ($M^{m+}$ ions) but in the form of aqua complexes $M(H_{2}O{)}_{n}^{m+}$, which, when incorporated into the chitosan structure or its modifier, exchange water ligands for others, forming more complex chelates for which a certain thermodynamic equilibrium is established.

In most procedures for preparing chitosan-based materials, chitosan is initially dissolved in an acetic acid solution. Next, the template ion is added together with modifiers and crosslinking agents. This leads to the formation of metal acetate complexes $M(OAc{)}_{n}^{m+}$, as well as metal complexes with introduced ligands or crosslinkers. Although acetate complexes have low stability constants and thus may have a negligible effect on ion-imprinting, complexes formed with crosslinkers or specific ligands significantly influence the properties of the final material [100]. The choice of the metal salt is also noteworthy. Nitrate, chloride, or sulfate salts, which dissolve readily in water, can form more stable complexes that may not effectively coordinate with ligands, resulting in weaker imprinting [101]. The conditions under which the printing process is carried out, such as pH, temperature, and solution ionic strength, are also very important, as they can alter the properties of the complex [102]. Regardless of the chosen method, the precise form the metal takes during this process is difficult to determine. This fact is crucial for preparing an ion-imprinted material and for explaining the imprinted sorbent’s increased sorption capacity compared to a sorbent prepared in the same way without the template ion.

Coordination chemistry, therefore, plays a major role in shaping and interpreting experimental results and can help elucidate the mechanism by which metal ions are removed using ion-imprinted sorbents. To facilitate the interpretation of sorption mechanisms using coordination chemistry, we present below a table of the most important parameters that may be useful for comparing experimental results and interpreting imprint formation (Table 3).

The ion-imprinting mechanism is influenced not only by the properties of the metal ions themselves but also by the complexes they may form with modified chitosan [103]. Mechanistically, metal uptake via sorbent involves multiple processes: protonation of amino and other charged groups via ionic interactions, chelation through coordination bonds, and stabilization by weak van der Waals forces [104].

Despite the information presented above, they exhibit different selectivity toward the template ions, due to the addition of template ions during synthesis (results in an imprinted sorbent) or not (non-imprinted sorbent), attributed to the role of the crosslinking agent, which not only contributes to network formation but also creates cavities of varying size and “locks in” the active sites where a metal ion can bind. This step is crucial, and the choice of an appropriate crosslinking agent can significantly influence the selectivity of the resulting sorbent. The most commonly used crosslinkers are GLA, ECH, and STPP [105]. Each exhibits a different binding mechanism and produces cavities of varying dimensions, which directly affect the sorbent’s properties. Also, the cross-linking agent can introduce an additional functional group to the polymer, which can form a coordination bond in a chelate.

Selecting an elution agent is also critical. In the case of chitosan, diluted inorganic acids (HNO_3_, HCl, and H_2_SO_4_) or ligands (such as EDTA) are typically used to remove the metal ions from the sorbent via complexation. It is important not only to select the appropriate elution agent but also to determine its concentration. At high acid concentrations, chitosan may dissolve or undergo structural changes associated with the degradation of crosslinking bonds, whereas at low concentrations, incomplete removal of template metal ions may occur, resulting in reduced selectivity or even complete loss of selectivity.

**Figure 6:**
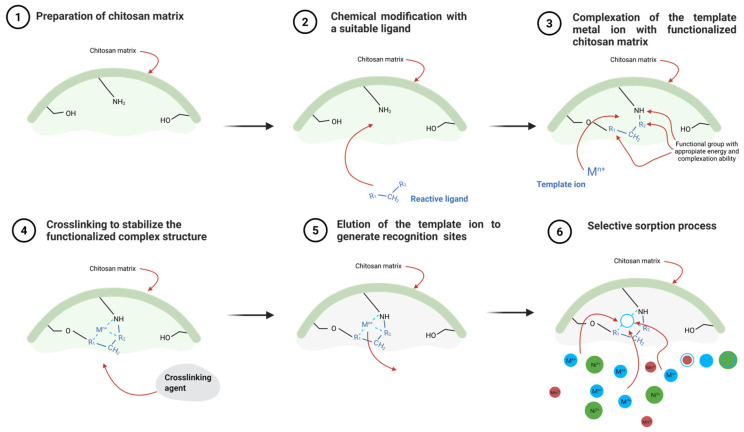
Proposed mechanism of ionic imprinting formation by chemical immobilization using chitosan. Source: Own research.

In summary, the ion-imprinting mechanism primarily involves the formation of stable chelates within the sorbent. The choice of ligands and modifiers is essential to ensure that the resulting complexes have appropriate thermodynamic stability: if the complex is too weak, the selectivity and imprinting efficiency are low; if it is too strong, the template ion cannot be easily eluted, preventing the production of a sorbent ready for efficient metal ion sorption. To ensure reliable evaluation of the imprinting effect, all steps in the procedure must be performed in the same manner for the non-imprinted sorbent (reference material). Particular attention should be paid to the removal of template ions step with the elution agent, as this may alter the sorption properties of the material. Below, we present a proposed mechanism for creating selective sites, based on a literature review (Figure 6).

## 7. Metal Ion Removal Using IIC Sorbents

**Figure 7:**
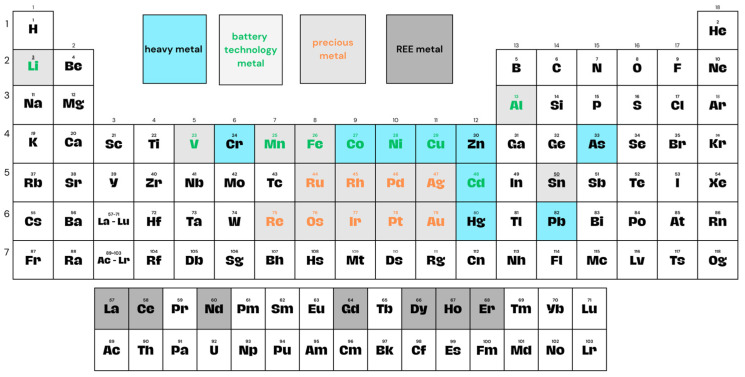
The periodic table groups of elements as heavy metals, battery technology metals, precious metals, and REE metals. Elements highlighted in blue indicate those discussed in this article. Source: Own research.

In this section, we discuss achievements in the field of ion metal removal from water and wastewater, with a focus on IIC-based sorbents. We specifically consider current and innovative methods and compare the sorption capacity, metal ion removal effectiveness, and selectivity of modified chitosan sorbents. The goal is to provide a framework on ion-imprinting in chitosan to support researchers by providing essential information regarding the techniques and modifications applied to chitosan. We believe that organizing the articles by relevant metals enhances clarity, and any existing gaps in the study of chitosan will be found and addressed by researchers in future publications. Due to the length of the manuscript, we present the review based only on heavy metal ions, which are marked in blue (Figure 7).

The presence of heavy metals, such as copper, lead, mercury, and nickel, as well as metal oxygenate anions—chromate and arsenate—poses a serious threat to human life and the environment [106]. While mining and volcanic dust are major sources of heavy metal production, human activities, for example, dyeing, metal plating, and battery manufacturing, also contribute to releasing these harmful substances [107]. Long-term exposure to heavy metals has resulted in decreased reproductive capacity in aquatic animals and respiratory and nervous problems. Furthermore, the accumulation of these metals in the body (biomagnification) and their transfer to the next consumers in the food chain, including humans, can lead to irreversible side effects [108]. This subsection is organized around selected metals that currently attract the greatest attention due to their environmental and industrial relevance.

### 7.1. The Removal of Chromium (Cr)

Chromium contamination in aquatic environments originates from both natural and anthropogenic sources, with chromium occurring predominantly as Cr(III) and Cr(VI), depending on pH and redox conditions [109]. Despite chromium’s environmental relevance, ion-imprinted chitosan-based sorbents (IICs) remain surprisingly underexplored, with only five studies reported to date, half of which focused on Cr(VI) removal.

A renewed interest in Cr(VI) ion-imprinting was reported in 2025 with the use of STPP as a flocculating and cross-linking agent [110]. The resulting chitosan-based hydrogel exhibited a porous structure and accessible functional groups, enabling effective chromium uptake. Furthermore, the authors showed that during the preparation of imprinted sorbents, the choice of eluent and its concentration, as well as the sorbent drying conditions, significantly influence the sorption properties. The imprinted material demonstrated a higher sorption capacity than its non-imprinted analogue (41.3 vs. 26.0 mg/g), indicating enhanced affinity toward chromium ions. Although selectivity was not explicitly quantified, the authors proposed a multistep removal mechanism involving electrostatic attraction, partial reduction of Cr(VI) to Cr(III), and subsequent surface complexation—an interplay that likely underpins the superior performance of the imprinted system. The use of a model solution containing not only chromium ions but also other ions and even organic compounds is also noteworthy.

In contrast to Cr(VI)-oriented systems, two studies have focused on the selective removal of Cr(III) via chelation-driven imprinting strategies. In 2024, chemically modified chitosan bearing nitrogen-rich donor groups showed high affinity toward Cr(III), achieving sorption capacities approaching 386 mg/g across a broad pH range [111]. High sorption capacity may be due not only to the use of a ligand containing a large amount of nitrogen in the ring, but also to the use of ECH in the imprinting process. The use of this cross-linking agent results in a high sorption capacity, even for sorbents without imprinting (290 mg/g). However, the disadvantage of this solution is the high toxicity of the cross-linking agent. Competitive sorption experiments confirmed preferential binding of Cr(III) over other transition metal ions, attributed to cooperative coordination involving amino and hydroxyl functionalities.

A similar chelation-enhanced approach was reported in 2022, where aromatic and phenolic groups were grafted onto the chitosan backbone to tailor the coordination environment for Cr(III) ions [112]. However, at this time, the material was cross-linked using glyoxal and the EDC/NHS coupling agent. This material exhibited a threefold increase in sorption capacity compared to the non-imprinted sorbent (250 vs. 90 mg/g) and maintained high selectivity even in complex multi-ionic solutions containing six competing metal ions, highlighting its potential relevance for real wastewater systems. Selectivity is explained by the formation of an active site after cross-linking, with a shape suitable for chromium(III) ions, due to the presence of a nitrogen atom from an -N=N- group and an oxygen atom from the phenolic group -OH.

Finally, an earlier study demonstrated that even non-classical imprinting approaches can yield remarkable results. An electrospun chitosan–graphene oxide composite showed an exceptionally high Cr(VI) sorption capacity exceeding 550 mg/g, along with clear selectivity next to Pb(II), Cu(II), and Ni(II) ions [113]. However, the high sorption capacities were associated with the use of nanofibers, which have large specific surface areas and easily accessible active sites. Although the imprinting mechanism in this case deviated from conventional approaches, the study highlights the importance of structural engineering in developing high-performance chromium sorbents. The imprinted sorbent was prepared using a modified procedure. Typically, the synthesis of ion-imprinted materials involves mixing the metal ion with the functional monomer, followed by polymerization (or, in the case of chitosan, cross-linking). In the present work, however, the material was first cross-linked, and only then was chromium introduced, sorbed onto the prepared matrix, and subsequently removed using a nitric acid solution. Afterwards, the sorbent was rinsed with water and dried. This raises the question of whether the resulting material can truly be classified as a chromium-imprinted sorbent, or rather whether the observed behavior may be attributed to an as-yet unidentified ‘memory effect’?

### 7.2. The Removal of Cobalt (Co)

Cobalt is a naturally occurring trace element essential for biological processes; however, elevated concentrations in aquatic environments pose serious ecological and health risks [114,115,116]. Anthropogenic activities such as mining, metallurgy, and improper waste disposal are the main sources of cobalt contamination in water systems. Excess cobalt can disrupt aquatic ecosystems, alter microbial communities, and bioaccumulate along the food chain, leading to toxic effects in higher organisms [117]. Despite the environmental relevance of cobalt, ion-imprinted chitosan-based sorbents (IICs) designed for its selective removal remain relatively underexplored. To date, only five studies have been reported, with research activity peaking between 2010 and 2022, and only a single recent contribution published in 2022.

The most recent study demonstrated the use of a thiourea-functionalized chitosan IIC for cobalt removal, showing rapid sorption kinetics and good performance under dynamic flow conditions [118]. Although selectivity was evaluated primarily against alkaline earth metal ions rather than competing transition metals, the study highlighted the practical applicability of ion-imprinted sorbents in real water matrices, where calcium and magnesium are dominant. The use of double printing is a major innovation here. At the same time, a sorbent was produced by adding two metal ions: cobalt and manganese, thereby increasing the sorption efficiency of these metals. This solution can be used when the interest is not in recovering a specific metal, and the group of removed metals can be used together as a mixed product. Of course, it is possible to separate these metals at subsequent stages, e.g., during selective elution. The use of thiourea increased sorption due to its complexing properties.

Earlier work emphasized ligand-assisted imprinting strategies to enhance cobalt selectivity. In one such study, the incorporation of nitrogen- and sulfur-containing ligands (8-hydroxyquinoline) into a chitosan matrix with Fe_3_O_4_ and EPI addition significantly improved Co(II) uptake, yielding sorption capacities substantially higher than those of non-imprinted materials (100 vs. 67 mg/g) [119]. Kinetic and thermodynamic analyses indicated a chemisorption-controlled process, underscoring the importance of tailored coordination environments in cobalt recognition.

In contrast, ligand-free imprinting approaches have also been explored, particularly for niche applications such as the removal of cobalt impurities from nuclear reactor systems [89]. These materials exhibited improved selectivity for Co(II) in the presence of large excesses of Fe(II), unfortunately, at the expense of very slow sorption kinetics, with equilibrium times extending to several days. Such limitations may restrict their applicability in conventional water treatment processes. In this work, EPI was also used for crosslinking. The overall sorption capacity of the modified material was much lower than that of unmodified chitosan, but the selectivity for cobalt ions increased, which is often a problem in ion-imprinting technology.

One of the earliest studies employed surface ion-imprinting on a chitosan–attapulgite composite, illustrating the benefits of inorganic and natural supports in enhancing sorption performance [120]. Despite imprinting, the resulting material exhibited a relatively low sorption capacity of 31.5 mg/g. Nevertheless, the imprinted sorbent exhibited a significantly higher distribution coefficient than its non-imprinted counterpart, clearly confirming the effectiveness of surface imprinting in enhancing selective cobalt recognition. In this study, a non-traditional crosslinking agent was used instead of the more toxic, commonly applied GLA or ECH, namely glycidoxypropyltrimethoxysilane (KH-560), an epoxy–siloxane compound containing trimethoxy anchoring groups.

Another even older publication that also used surface imprinting dates back to 2009 and employed KH-560 as a crosslinking agent [121]. Lower sorption capacities of 22 and 8 mg/g were obtained for the imprinted and unimprinted sorbents, respectively. Instead of attapulgite as an inorganic filler, they used potassium titanate whiskers. Importantly, the material showed high selectivity for metal ions, with the highest relative selectivity for nickel ions, which is a very good result considering the similarity between these two metals.

Overall, the limited number of studies and their strong methodological diversity indicate that cobalt-selective IICs remain an underdeveloped research area. Existing reports nevertheless demonstrate that both ligand-assisted and ligand-free imprinting strategies can achieve enhanced selectivity toward Co(II), although challenges related to kinetics, scalability, and comprehensive selectivity evaluation persist. These gaps suggest significant opportunities for the further development of chitosan-based ion-imprinted materials targeting cobalt removal.

### 7.3. The Removal of Nickel (Ni)

Nickel is widely used in various industries, including metallurgy, electroplating, and battery production [122]. The accumulation of nickel in living organisms is a significant concern, as it is not biodegradable and can lead to health problems such as lung cancer, cardiovascular disease, and neurological deficits [122]. The presence of nickel and its compounds in water resources can also adversely affect aquatic ecosystems, disrupting the natural balance and harming various plant and animal species [123,124].

Researchers have explored sustainable and effective methods for removing nickel ions from wastewater and water, including the use of IIC. To date, more than a dozen studies have reported the successful application of chitosan-based ion-imprinted sorbents for selective Ni(II) removal. Notably, most of these publications are relatively recent, with the majority appearing after 2018. Interestingly, research activity in this area appears more extensive than that devoted to cobalt removal, despite cobalt being considered more critical and strategically important. Selected representative studies on nickel ion-imprinting using chitosan are discussed below.

Early studies demonstrated that the incorporation of magnetic nanoparticles into chitosan matrices, cross-linked with STPP, enhances both selectivity and operational convenience [125]. Such materials typically show stronger discrimination against Zn(II) than Cu(II), reflecting the close chemical similarity between Ni(II) and Cu(II). The sorption capacity using the Langmuir model was 18.5 mg/g, about 8 times higher than for the non-imprinted sorbent. Although the reported sorption capacities remained moderate in some cases, imprinting consistently led to multi-fold improvements over non-imprinted counterparts, confirming the effectiveness of the approach.

A major research trend involves the use of composite and hybrid materials to boost sorption performance. Waste-derived chitosan composites incorporating activated carbon, melamine, or biomass additives have attracted attention due to their alignment with circular economy principles [126]. Importantly, this composite is obtained from waste only (from human hair and shrimp shell waste). These systems frequently exhibit sorption capacities exceeding 100 mg/g and relative selectivity coefficients around 5, clearly outperforming non-imprinted analogues.

Another study investigated the use of chitosan crosslinked with ECH [127]. Unfortunately, the resulting material exhibited a low sorption capacity of only 29.5 mg/g, but satisfactory selectivity coefficients of approximately 50. The study did not include a material prepared without nickel, which would have allowed for a comparison between imprinted and non-imprinted sorbents.

Surface ion-imprinting strategies and chemically modified chitosan derivatives have further expanded the performance window of nickel-selective sorbents. Several studies reported sorption capacities of 80–135 mg/g, accompanied by good selectivity against competing ions such as Co(II), Cu(II), Cd(II), and Pb(II) [128,129,130]. In some cases, particularly high discrimination against cobalt was achieved despite its close ionic radius and coordination behavior being close to those of nickel. Importantly, such materials have been successfully applied to practical systems, including waste battery leachates, demonstrating high nickel removal efficiencies and effective separation from cadmium.

Another notable development is the incorporation of carbon allotropes, especially carbon nanotubes, into chitosan-based IICs [131]. These composites benefit from increased surface area and improved mass transfer, often reaching sorption equilibrium within minutes. Enhanced sorption capacities (above 50 mg/g) and stable reusability over multiple cycles further underline the advantages of this strategy.

In addition to chemical modifications, the sorbent’s physical form also plays a significant role in performance. Chitosan foams, microspheres, pellets, and beads have all been explored, with porous structures generally offering higher accessibility of imprinted sites. In particular, foam-like and bead-based materials have demonstrated good mechanical stability, moderate-to-high sorption capacities (around 70 mg/g), and consistent selectivity in both batch and dynamic systems [132].

Building on these advances in composition and form, ion-imprinted chitosan materials have also proven effective in solid-phase extraction (SPE) and trace analysis applications [133]. Magnetic IICs, in particular, enable the efficient preconcentration of nickel from complex water matrices containing high concentrations of competing ions. Reported nickel recoveries above 90% in multi-ionic environments underscore the analytical potential of these systems.

Taken together, these developments reveal a broad scope for chitosan-based materials. Across the literature, reported maximum sorption capacities span a broad range—from below 20 mg/g to as high as 500 mg/g—reflecting the strong influence of composite design, imprinting strategy, and material architecture [134,135]. Most studies confirm good reusability, typically maintaining performance over at least five to ten sorption–desorption cycles. Overall, these findings highlight chitosan-based ion-imprinted materials as a versatile and highly promising strategy for the selective removal of Ni(II) ions from water.

### 7.4. The Removal of Copper (Cu)

Copper pollution in water poses a significant environmental concern due to its toxicity at high concentrations [136]. Industrial activities, mining, and agricultural practices contribute to copper contamination in soil and water [137]. Excessive exposure to copper can lead to severe health issues, including liver damage, kidney disorders, and anemia [138]. Various methods have been developed to detect and remediate copper pollution in water samples, including spectrophotometry, mass spectrometry, sensors, voltammetry, chromatography, and bioremediation using microorganisms [138,139]. While physical and chemical approaches can be effective, they often entail high costs and produce sludge [136]. Researchers are also exploring environmentally friendly sorption processes to extract copper compounds from wastewater and to promote sustainable water reuse.

Recent studies have yielded a considerable number of publications on the synthesis and application of copper IIC sorbents. More than twenty articles on this topic were identified and described below. The strong interest in the removal of copper(II) ions using chitosan may be related to its very high removal efficiency, which results from the chelation of copper ions by free amino groups.

Oxide nanoparticle–chitosan-based hydrogels represent another intensively investigated class of Cu(II)-imprinted sorbents [140,141,142,143]. By incorporating iron oxide or aluminum oxide nanoparticles, these materials enable rapid separation while maintaining good sorption performance. Studies consistently demonstrate that copper imprinting leads to higher affinity and selectivity than non-imprinted counterparts, as well as good stability across multiple sorption–desorption cycles. These features make oxide-particle IIC hydrogels particularly attractive for repeated use in practical water treatment applications.

For hydrogels modified with iron oxide, maximum sorption capacities of 85.1, 100.3, and 46.3 mg/g were reported [140,142,143], whereas for aluminum oxide-modified sorbents, a value of 31.4 mg/g was obtained [141]. Naturally, these sorbents were synthesized under different conditions and procedures, making direct comparison difficult. The high sorption capacity reported in work [142] may be attributed to the sorbent being prepared as microspheres, which significantly increased the surface area.

Porous chitosan materials, including microporous structures, cryogels, and microspheres, have also been explored as platforms for copper ion-imprinting [144,145,146,147]. Their interconnected pore networks facilitate efficient mass transfer and improve accessibility to imprinted binding sites. In some cases, incorporating natural additives, such as zeolites, further enhances sorption efficiency while preserving selectivity. For these studies, the maximum sorption capacities calculated using the Langmuir model were 82.6 mg/g [144], 261.3 mg/g [145], 82.0 mg/g [146], and 201.7 mg/g [147]. For the cryogel sorbent, an increase in selectivity for copper ions was apparent, with selectivity coefficients of 4.1, 4.6, 1.9, and 11.3 for Zn(II), Ni(II), Fe(III), and Cr(III), respectively, in binary systems [144]. These studies provide valuable mechanistic insights, linking copper preference to ion speciation, coordination behavior, and the physicochemical properties of competing metal ions.

Chemical modification of chitosan through Schiff-base formation, Mannich reactions, or ligand grafting remains one of the most effective strategies for strengthening copper recognition [148,149,150]. The introduction of additional donor atoms creates coordination environments well-suited to Cu(II), and imprinting within such matrices typically results in a pronounced contrast between imprinted and non-imprinted materials. For example, sorption capacities increased from 37 mg/g to 143 mg/g and from 27.4 mg/g to 163.1 mg/g for ion-imprinted sorbents modified with isatin and 8-hydroxyquinoline, respectively [148,149]. Many of these sorbents also exhibit good regeneration capability, retaining high performance over several sorption–desorption cycles.

Most sorbents use ECH or GLA as crosslinking agents. A study reported an alternative approach using bisphenol A diglycidyl ether (BADGE) for crosslinking [151]. However, this agent did not yield favorable results. Unmodified chitosan showed higher sorption capacity and selectivity (17.5 vs. 6.5 mg/g). The low sorption capacity may be due to the limited availability of free amino groups, which effectively complex copper ions, and to the lack of functional groups in the crosslinker that could help form copper-selective binding sites. This topic would require more in-depth investigation.

Moving from single-polymer systems, composite materials combining chitosan with minerals or other biopolymers further demonstrate the flexibility of the imprinting concept [152,153,154]. For sorbents modified with montmorillonite, sodium alginate, and PVA, sorption capacities of 119.4 mg/g, 83.3 mg/g, and 232.6 mg/g were achieved, respectively. Although the degree of selectivity enhancement varies depending on material structure, imprinting consistently shifts sorption behavior toward preferential copper binding. Even simplified synthesis routes frequently benefit from the imprinting step, underscoring its decisive role in defining copper affinity.

Building on these advances, hybrid hydrogel systems incorporating both organic and inorganic components have reported some of the highest copper sorption capacities among Cu(II)-imprinted materials [155,156]. The maximum Langmuir sorption capacities were 259.6 mg/g for the imprinted material and 219.6 mg/g for the non-imprinted material, clearly demonstrating the enhancement in sorption selectivity due to imprinting [155]. Competitive sorption experiments confirmed strong selectivity against commonly coexisting metal ions, while recyclability tests demonstrated excellent mechanical stability and sustained performance over multiple cycles [155]. In the second work, the sorbent was synthesized using iron oxide nanoparticles together with graphene oxide (GO), achieving 142.9 mg/g [156].

Taken together, these findings clearly demonstrate that Cu(II) ion-imprinting on chitosan is a robust and adaptable strategy for selective copper removal. Across diverse material forms—including nanofibers, hydrogels, cryogels, and composites—the imprinting approach consistently enhances copper affinity, improves selectivity in multicomponent systems, and enables material reuse. These attributes position chitosan-based IIC sorbents as promising candidates for sustainable copper remediation, recovery, and monitoring in complex aqueous environments.

### 7.5. The Removal of Zinc (Zn)

Zinc pollution in water is a significant environmental concern with potential health risks. Excessive zinc levels in drinking water can lead to respiratory, liver, and brain disorders [157]. Sources of zinc contamination include industrial effluents, corrosive pipelines, and mining activities [157,158]. Studies have shown that zinc concentrations in bodies of water can exceed the permissible limits set by health organizations [158]. Acute zinc toxicity is rare, with symptoms such as nausea and fatigue occurring only at extremely high intakes [159,160]. However, prolonged high-dose zinc supplementation can interfere with copper uptake, leading to copper deficiency and associated symptoms [160].

Given these health and environmental concerns, it is important to evaluate relevant remediation strategies. Only two articles on chitosan sorbents for zinc ion-imprinting were found in the databases; therefore, this section discusses them in detail.

The first study, published in 2020, explored zinc ion-imprinting as a selective strategy for Zn(II) extraction from aqueous media using methacrylamide-based copolymerization [161]. The significance of this study lies in its demonstration of an approach that achieves high removal efficiencies (91–99%) for zinc in real water samples, despite a modest maximum Langmuir sorption capacity (3.1 mg/g). By focusing on environmentally relevant zinc concentrations below 1 mg/dm^3^, the work underscores applicability to real-world scenarios, rather than purely idealized conditions. The lack of comparison with a non-imprinted material limited the assessment of the true imprinting effect, suggesting that further studies are needed to fully establish the method’s selectivity.

In 2017, researchers reported a multifunctional sorbent by combining chitosan with graphene oxide and magnetite nanoparticles for a magnetically recoverable Zn(II)-imprinted composite [162]. Graphene oxide enhanced surface functionality and magnetite enabled rapid separation with an external magnetic field. Zinc imprinting used zincon as a selective ligand, creating a material with high sorption capacity (71.4 mg/g) and fast kinetics, reaching equilibrium in 150 min. Zinc removal was optimal at pH 8.0, matching favorable Zn(II) speciation and ligand coordination. This sorbent also recovered over 97% of zinc from complex food matrices such as milk, black tea, and rice. The material maintained high extraction efficiency through at least ten sorption–desorption cycles. These features make the graphene–magnetite–chitosan Zn(II)-imprinted composite a robust, scalable option for zinc monitoring and removal in environmental and food applications.

Few publications have focused on selective zinc removal. This research gap exists because zinc is often seen as not requiring intensive remediation. However, the widespread use of zinc in cementation leads to significant consumption and potential environmental releases. Targeted research is needed to develop and access methods for selective zinc removal to mitigate these environmental impacts.

### 7.6. The Removal of Arsenic (As)

Arsenic occurs naturally in the environment in various forms, with arsenite and arsenate being the main forms in soil [163]. Its pollution is a global concern, particularly in South Asia, West-Central Africa, Western Europe, and Latin America [164]. Arsenic contamination in water and soil can result from natural weathering, industrial activities, and mining [165]. The toxicity of arsenic depends on its form, with inorganic arsenic being more toxic than organic forms [166]. Arsenic pollution poses severe health risks, including cancer and other ailments [164]. The uptake and accumulation of arsenic in plants vary depending on factors such as soil concentration and plant species [167]. To address these issues, sustainable remediation technologies like sorption, constructed wetlands, and phytoremediation have been proposed [164]. Arsenic occurs in nature in inorganic form mainly in two oxidation states, As(III) and As(V). Our literature review focused on the sorption of these two forms and identified five publications.

The most recent study, published in 2023, focused on microwave-assisted imprinting of As(V) ions [168]. The sorbent was obtained by pre-crosslinking chitosan with GLA in the presence of As(V), followed by functionalization with TiO_2_ and SiO_2_ nanoparticles to enhance surface reactivity and mechanical stability. The resulting hybrid material exhibited a remarkably high Langmuir sorption capacity of 625 mg/g. Selectivity tests performed in the presence of competing Cu(II), Ca(II), Mg(II), K(I), and Na(I) ions yielded selectivity coefficients of 4.35, 11.20, 5.28, 5.32, and 6.20, respectively. Notably, adsorption equilibrium was achieved within only 20 s, attributed to microwave irradiation facilitating rapid mass transfer and greater accessibility of binding sites. The sorbent maintained satisfactory performance for up to three sorption–desorption cycles, confirming its short-term reusability.

In contrast, the most recent work addressing As(III) imprinting dates back to 2020 and employed a considerably more complex Pickering emulsion polymerization strategy combined with ultrasonic-assisted micro solid-phase extraction (UA-µ-SPE) [169]. The synthesis involved multiple functional components, including a thiosemicarbazone ligand, magnetic nanoparticles, and a chitosan colloidal stabilizer, resulting in a magnetically separable imprinted composite. Despite the elaborate preparation, the imprinted sorbent showed a clear imprinting effect, with a Langmuir capacity of 37.04 mg/g compared with only 1.25 mg/g for the non-imprinted analogue. The relative selectivity coefficient reached 29.44, confirming a strong preference for As(III). Sorption behavior was mainly governed by pH, sorbent dosage, and ultrasonication time. The material retained its performance for five cycles and enabled nearly quantitative arsenic recovery from real samples, including water and plant-based matrices, demonstrating its practical applicability.

Earlier studies from 2011 by the same research group explored simpler bead-type chitosan resins prepared via suspension crosslinking [170,171]. In these systems, arsenic-loaded chitosan was crosslinked with GLA to form spherical particles, followed by template removal. Although the preparation was straightforward and scalable, the sorption capacity was relatively low (4.4 mg/g). The distribution ratio of the imprinted sorbent was approximately 4 times that of the non-imprinted material, and the beads could be reused up to 5 cycles with a 20% efficiency loss. These resins were also successfully applied in column systems and for arsenic removal from juice samples, highlighting their potential for practical separations despite limited capacity.

The oldest identified work on As(III) imprinting further modified chitosan beads with α-Fe_2_O_3_ to introduce additional active sites and improve affinity [172]. This composite exhibited a higher Langmuir capacity of 9.4 mg/g and exceptional selectivity in the presence of Fe(III), with a relative selectivity coefficient of 502.3, suggesting strong synergistic interactions between iron oxide and arsenic species. The sorbent maintained acceptable stability over 10 cycles, with only a 20.2% decrease in efficiency, and was proposed for the solid-phase extraction of iron-rich samples.

Overall, the available studies indicate that arsenic imprinting on chitosan remains underexplored compared to other heavy metals. While advanced hybrid and nanostructured systems can achieve very high capacities and rapid kinetics, simpler bead-type materials offer easier synthesis and practical applicability at the expense of lower performance. Given the high toxicity and environmental relevance of arsenic species, the limited number of reports underscores a research gap and highlights the need to further develop selective, stable, and reusable chitosan-based imprinted sorbents. It would be interesting to develop a chitosan-based material for the separation of these two speciation forms, for example, using solid-phase extraction (SPE).

### 7.7. The Removal of Cadmium (Cd)

Cadmium is another heavy metal pollutant that poses a significant threat to human health. Cadmium is ranked as the seventh most toxic heavy metal, and it can easily enter the food chain and accumulate in the human body over time [173]. Exposure to cadmium can harm the heart, lungs, bones, and especially the kidneys, making it a major public health concern [174]. This metal is commonly used in various industrial processes, such as electroplating, battery production, and pigment manufacturing, leading to its accumulation in bodies of water [175]. As industrialization has increased, the issue of cadmium pollution has become increasingly severe, with the metal being found in plastic toys, batteries, paints, ceramics, and even cigarette smoke [176]. According to the available literature, more than a dozen publications have addressed the imprinting of cadmium onto chitosan.

The most recent publication discussed the potential use of STPP to prepare imprinted chitosan [177]. The study presents a description of the mechanism and a discussion of the order of metal affinity toward the sorbent, which was attributed to differences in the ionic radii of the metals in the following order: Cd(II) (0.95 Å) > Co(II) (0.74 Å) > Ni(II) (0.71 Å). The maximum sorption capacity reached 1.06 mmol/g, and the work is largely based on the authors’ previous study [178]. STPP-crosslinked chitosan displayed capacities of 1.05 mmol/g (IIP) versus 0.87 mmol/g (NIP), with limited mechanistic evidence for a strong imprinting contribution [178].

Recent reports on Cd(II)-imprinted chitosan materials reveal a clear shift from conventional hydrogel sorbents toward multifunctional composites that integrate sensing capability, nanostructuring, or hybrid organic–inorganic structures to enhance selectivity and operational robustness. The most recent study (2024) demonstrated that a chitosan/sodium alginate hydrogel acts simultaneously as a sorbent and colorimetric sensor, achieving very high Langmuir capacities of 534.9 mg/g for the imprinted material and 324.7 mg/g for the non-imprinted analogue, along with good stability over multiple cycles [179]. Although equilibrium was relatively slow, the pronounced imprinting effect and clear visual response underline the growing interest in dual-function materials that combine detection with removal.

Nanocomposite and magnetically assisted systems represent another important direction. Chitosan-coated MnFe_2_O_4_/graphene oxide hybrids enabled the simultaneous imprinting of Cd(II) and Ni(II), offering capacities of 45.87 mg/g (IIP) and 30.39 mg/g (NIP) and convenient magnetic separation with good reusability [180]. Likewise, earlier magnetic sorbents based on modified chitosan and Fe_3_O_4_ nanoparticles showed a more pronounced imprinting benefit, with 26.1 mg/g for the imprinted material compared with only 6.7 mg/g for the non-imprinted one [181]. Such systems emphasize improved mass transfer and facile recovery rather than the maximization of absolute capacity, which is advantageous for practical water treatment.

Chemical functionalization of chitosan with chelating ligands remains one of the most effective strategies to enhance both affinity and selectivity. Thiosemicarbazide-modified chitosan exhibited high performance, reaching 305 mg/g for the imprinted sorbent versus 158 mg/g for the non-imprinted counterpart, accompanied by strong discrimination against competing divalent metals [182]. Similarly, grafted or crosslinked polymeric networks prepared for real-sample applications, such as wastewater from Ni–Cd batteries, achieved capacities of 167 mg/g (IIP) and 71.4 mg/g (NIP), confirming the practical relevance of tailored binding sites [183].

Surface-imprinted composites based on waste oyster shells reached 70.5 mg/g, although no non-imprinted reference was reported, making the imprinting effect difficult to assess quantitatively [184]. These examples suggest that straightforward formulations may offer easier synthesis but often at the expense of selectivity or clear structure–performance relationships.

Earlier works laid the foundation for current developments using diverse suspension or surface polymerization strategies. Dual-template systems prepared by suspension polymerization achieved selective capacities of 48 mg/g (IIP) and 12 mg/g (NIP) with a Langmuir capacity of 38.6 mg/g for the imprinted sorbent [185], demonstrating that even relatively simple imprinting approaches can provide measurable selectivity gains.

In summary, the literature demonstrates that Cd(II) ion-imprinting on chitosan is a versatile and effective strategy for selective cadmium capture. Across a range of material formats—including hydrogels, magnetic nanocomposites, chemically functionalized derivatives, and bio-based supports—imprinting consistently improves metal affinity and selectivity in multicomponent systems. However, significant variability in reported performance underscores the influence of ligand chemistry, porosity, and mass-transfer properties. Future research should prioritize not only on increasing capacity but also simplifying synthesis, enhancing kinetics, and validating performance in real, complex matrices. These advancements could establish chitosan-based ion-imprinted composite IIC sorbents as practical solutions for sustainable cadmium remediation and resource recovery.

### 7.8. The Removal of Mercury (Hg)

Mercury is a toxic element that poses significant risks to human health and the environment [186]. Present in various forms, including inorganic and organic compounds, mercury can accumulate in tissues and organs, particularly in the brain, kidneys, and liver [187]. Notably, the literature on mercury imprinting on chitosan comprises only two articles. As with studies on zinc, these are discussed in greater detail.

Building on these findings, a 2024 publication described a DNA-functionalized chitosan composite designed as an electrochemical sensor for Hg(II) detection in aqueous media [188]. The material was prepared by forming a chitosan–mercury complex, crosslinking with STPP, and depositing the resulting complex onto a carbon electrode to create a thin sensing membrane. After EDTA-assisted template removal, the surface was further modified with a DNA-based enzymatic layer and 6-mercaptohexan-1-ol to enhance recognition and signal transduction. Owing to the synergistic effect of molecular imprinting and DNA affinity toward mercury, the sensor exhibited high selectivity for Hg(II) even in the presence of competing ions such as Ag(I), Pb(II), Fe(III), Mn(II), and Ca(II). Extensive physicochemical characterization confirmed the stability and reproducibility of the sensing platform, highlighting its potential for rapid and selective mercury monitoring in water samples.

In contrast to the sensor-focused approach, earlier work from 2022 concentrated on mercury removal rather than sensing, employing a ligand-modified chitosan sorbent for selective adsorption [189]. In this case, chitosan was functionalized with a Schiff-base ligand derived from 4-amino-3-hydroxybenzoic acid and 2-pyridinedicarboxaldehyde, introducing additional chelating sites with high affinity for Hg(II). After complexation with mercury ions and crosslinking with GLA, spherical microgranules were obtained and subsequently eluted to remove the template. The imprinted sorbent exhibited a high Langmuir sorption capacity of 315 mg/g, more than double that of the non-imprinted analogue (145 mg/g), clearly demonstrating the effectiveness of the imprinting strategy. Selectivity tests toward various competing metal ions revealed the strongest discrimination against Pb(II), with a relative selectivity coefficient of 17.9. Furthermore, the material maintained stable performance over at least five sorption–desorption cycles, confirming its reusability and practical applicability for mercury remediation.

Taken together, these two studies demonstrate the limited research activity in mercury imprinting, despite mercury being one of the most prevalent and hazardous heavy metals. This observation clearly indicates a research gap and highlights the need for further investigation in this area. Furthermore, while mercury shows a high affinity for unmodified chitosan, the material lacks selectivity, which could potentially be improved through imprinting techniques.

### 7.9. The Removal of Lead (Pb)

Lead toxicity is a serious public health problem [190]. Sources of lead exposure include paint, dust, water, soil, and traditional medicines [191]. Lead induces oxidative stress, disrupts cell membranes, and interferes with neurotransmitter levels, potentially causing hypertension, anemia, cognitive deficits, and organ damage [191]. The severity of lead-related health issues has sparked significant scientific interest, with more than 10 publications dedicated to the imprinting of lead onto chitosan-based materials.

Recent studies on Pb(II)-imprinted chitosan materials demonstrate a clear evolution from simple crosslinked beads toward multifunctional and hybrid sorbents that combine ion-imprinting with nanofillers, grafted polymers, or magnetic components to enhance selectivity, kinetics, and operational stability. For instance, nanocomposite systems incorporating TiO_2_/SiO_2_ nanoparticles enabled very rapid sorption and reached a Langmuir capacity of 81.97 mg/g for the imprinted material [168]. Similarly, grafted polymer networks based on styrene sulfonate and acrylic acid showed comparable performance, with capacities around 82 mg/g for the imprinted sorbent, although competitive ions such as Zn(II) significantly reduced uptake [192]. These studies highlight that surface functionalization and improved accessibility of binding sites are key factors governing fast and selective Pb(II) capture.

Other approaches focused on copolymerization or incorporation of magnetic or carbon-based additives to facilitate separation and practical use. CMC-based magnetic systems achieved a capacity of 124.07 mg/g while maintaining good selectivity toward competing Cu(II), Cd(II), and Ni(II) ions and proved effective for the solid-phase extraction of real water samples [193]. Copolymerized chitosan networks prepared with vinyl monomers showed lower capacities, with 30.12 mg/g for the imprinted sorbent versus 29.06 mg/g for the non-imprinted analogue, suggesting that the imprinting effect was more pronounced in kinetics than in equilibrium capacity [194]. In contrast, thermosensitive CNT-containing composites displayed a clearer imprinting benefit, with 83.2 mg/g for IIP compared to 26.06 mg/g for NIP, while also enabling temperature-controlled regeneration [195].

Several studies explored chemically modified chitosan derivatives, particularly carboxymethyl chitosan or blends with pectin. Although some systems reported high absolute capacities, such as 167.1 mg/g (IIP) versus 128 mg/g (NIP) [196], or improvements relative to raw polymers despite low overall uptake (0.66 mg/g) [197], the selectivity enhancement was not always consistent. In several cases, non-imprinted materials performed similarly, indicating that the mere presence of additional functional groups does not guarantee well-defined recognition sites and that the structural organization of the imprinted cavities remains crucial [198].

More unconventional strategies involved micellar or biologically assisted synthesis. Sorbents incorporating bacterial or micellar templates exhibited sorption capacities of 116.28 mg/g for the imprinted polymer (IIP) and 70.92 mg/g for the non-imprinted polymer (NIP) [199]. However, earlier systems demonstrated comparable uptake values of approximately 69 mg/g even for non-imprinted materials, suggesting only a limited imprinting effect [200]. These approaches emphasize improved morphology and accessibility rather than purely chemical selectivity.

Earlier foundational reports already demonstrated the potential of relatively simple chitosan systems. Crosslinked nanoparticles reached 74.07 mg/g without a direct NIP comparison [88] while diatomite-supported surface-imprinted beads achieved 139.6 mg/g, approximately 32% higher than the corresponding non-imprinted sorbent [201]. Together, these early studies established the feasibility of Pb(II) imprinting and provided performance benchmarks for later modifications.

Overall, Pb(II)-imprinted chitosan sorbents range from marginal to highly pronounced, depending on material architecture. The most promising systems balance capacity with fast kinetics, mechanical stability, selectivity, and easy regeneration. Current trends indicate a shift away from maximizing uptake alone. The goal now is application-oriented, multifunctional composites suitable for real-sample treatment.

The sorbents discussed in the text, and even more, are summarized below in Table 4.

## 8. Conclusions and Future Perspectives

The desirable properties of ion-imprinted chitosan (IIC) materials position them as promising candidates for water and wastewater remediation. Chitosan, a biopolymer derived from chitin, offers biocompatibility, biodegradability, and functional versatility, which make it particularly suitable for environmental applications. This review highlights the significant potential of IIC sorbents in water treatment, chemical sensors, analytical chemistry (e.g., solid-phase extraction), and the recovery of valuable metals from aqueous media, underscoring their promise as transformative solutions to current and future environmental challenges.

### 8.1. Summary and Conclusions

A comprehensive analysis of the literature on IIC sorbents yielded several key conclusions:

#### 8.1.1. Research Focus on Different Metals

**Figure 8:**
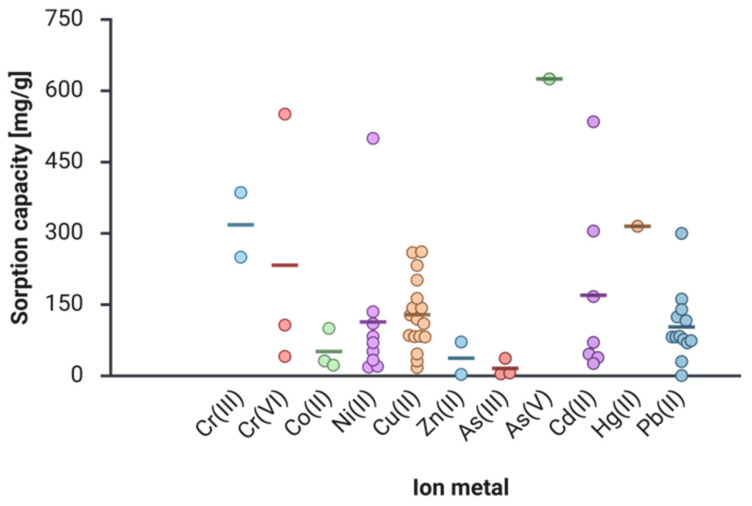
Comparison of sorption capacities reported in articles on heavy metals. The average of sorption capacity is indicated by “—”. Source: Own search.

Most existing studies focus on specific heavy metals. Copper has received the greatest attention, with approximately 20 publications, followed by nickel (11) and cadmium (8). Notably, cobalt has been the subject of only five studies, despite its critical status regarding supply risk and economic importance. Mercury has received even less attention, with only two publications, despite its severe and well-documented environmental and health impacts. This uneven distribution of research interest indicates a need to broaden the scope of future investigations to include environmentally hazardous or strategically significant metals (Figure 8). A key research gap is the development of novel materials with high selectivity toward Cr(III), Zn(II), As(V), and Hg(II). Investigating such sorbents may enable the effective remediation of wastewater containing these elements.

#### 8.1.2. Synthesis Approaches

The synthesis of IIC derivatives often involves Schiff-base reactions with carbodiimide-based coupling agents such as EDC/NHS. Although these reagents are effective, they are relatively expensive, thereby increasing the overall cost of sorbent preparation. Crosslinking is frequently achieved with ECH or GLA, both of which are highly toxic. However, the potential environmental impact of these reagents is primarily associated with their free (unreacted) forms. After crosslinking, they become incorporated into the chitosan network, which significantly reduces their mobility and bioavailability. Therefore, the use of such crosslinkers does not necessarily compromise the environmentally friendly nature of chitosan-based materials.

In this context, chitosan maintains its advantages as a natural, renewable, and biodegradable polymer. The overall sustainability of the final material depends on balancing its intrinsic properties with the selection and management of modifying agents. Rather than presenting a direct contradiction, this situation reflects a trade-off between material performance and environmental considerations. Increased attention should be directed to more environmentally friendly crosslinking agents, such as genipin, oxalic acid, STPP, or cellulose derivatives, which represent a notable research gap in this field.

EDTA solutions are widely used for metal-ion elution. However, thorough rinsing of sorbents after elution is essential, as residual EDTA can artificially increase the apparent sorption efficiency. This issue is particularly problematic when comparing imprinted and non-imprinted sorbents, since the reference materials are often not exposed to EDTA. In addition to EDTA, mineral acids such as HCl, HNO_3_, and H_2_SO_4_ are also effective eluents. However, traces of these acids may remain in the sorbent structure and alter its properties, for example, by shifting the internal pH relative to the external solution, thereby affecting sorption behavior. Rigorous washing protocols are therefore necessary to ensure reliable results and reproducibility.

### 8.2. Limitations and Future Perspectives

A recurring limitation in ion-imprinting research is the absence of non-imprinted reference sorbents in comparative experiments. Without such controls, attributing selectivity improvements to the imprinting effect is challenging. High-quality studies typically include comparative tables that explicitly present partition coefficients, selectivity coefficients, and relative selectivity values for imprinted versus non-imprinted materials. Broader adoption of these practices would significantly enhance the scientific rigor of future studies and enable more accurate benchmarking across research groups.

Despite significant progress in the development of IIC sorbents, several important limitations remain inadequately addressed in the literature.

First, many studies rely on simplified laboratory conditions that do not adequately reflect the complexity of real wastewater systems, which contain competing ions, organic matter, and fluctuating physicochemical parameters. As a result, the reported selectivity and adsorption capacities may be overestimated.

Second, the mechanisms of cavity formation and “energy matching” are often inferred indirectly, without direct structural or spectroscopic evidence, which limits the depth of mechanistic understanding.

Third, reproducibility remains a challenge, as small variations in synthesis conditions (e.g., pH, crosslinking efficiency, or template removal efficiency) can significantly affect sorbent performance.

In addition, insufficient attention is often paid to mass transfer limitations and diffusion constraints, particularly in highly crosslinked materials, which may reduce the accessibility of imprinted sites. Finally, the environmental impact of chemical modification steps, including the use of crosslinkers and eluents, has rarely been systematically evaluated. Addressing these limitations will be essential to translating laboratory-scale successes into reliable, sustainable real-world applications.

The increasing number of publications on IIC sorbents confirms that this is a rapidly developing research area. Most studies report enhanced efficiency and selectivity of IIC compared with conventional chitosan-based materials, underscoring their potential. However, practical applications in large-scale industrial or municipal wastewater treatment remain limited. Bridging the gap between laboratory-scale experiments and real-world systems is a pressing challenge.

A crucial future direction is the systematic testing of these sorbents in real wastewater matrices, which are chemically complex and may contain competing ions and organic matter. Computational modeling and artificial intelligence may assist in predicting sorption behavior under such conditions, optimizing synthesis routes, and guiding experimental design. Those involved in the synthesis of chitosan-based materials know that these experiments are very time-consuming. This necessitates the use of statistical methods to minimize the number of trials (e.g., by applying DOE experimental design in Statistica).

Another major task is to improve the crosslinking stage while preserving chitosan’s ecological advantages. The development of new, environmentally benign crosslinkers, derived, for example, from natural or renewable sources, could represent a breakthrough in making ion-imprinted sorbents fully “green” (such as genipin, or organic acids like citric acid, succinic acid, etc.). From an economic standpoint, cost assessments of sorbent synthesis are expected to become a standard requirement in high-impact journals, further stimulating research into affordable and scalable production methods.

To fully realize the potential of chitosan-based ion-imprinted sorbents, innovative synthetic approaches are needed. One promising avenue is 3D printing, which allows for precise control over sorbent geometry and porosity, enabling the design of tailor-made structures for purification devices. Such technologies could pave the way for customized sorbents optimized for specific applications, such as point-of-use water purification or selective recovery of strategic metals from industrial effluents.

Hydrogels based on chitosan represent another particularly attractive research direction. These materials exhibit tunable swelling, porosity, and mechanical properties that can be harnessed to overcome diffusion limitations and enhance sorption kinetics. In addition, chitosan hydrogels retain the inherent advantages of the biopolymer—biocompatibility, biodegradability, and renewability—while offering superior performance in sorption-based water treatment. Nevertheless, the introduction of imprinting functionalities through chemical coupling inevitably alters the biodegradability of the base polymer. More systematic studies are required to evaluate how various modifications affect the long-term environmental fate of chitosan-based sorbents.

The formulation of IIC materials often involves blending with other polymers or biopolymers, such as PVA or alginate. This approach opens new opportunities for composite materials that combine the strengths of different components. Future trends are likely to focus on composite hydrogels that offer enhanced stability, mechanical strength, and controlled release properties, extending their applicability to advanced separation processes and even biomedical or pharmaceutical uses.

Building on these developments, the modified chitosan ion-imprinted sorbent can be implemented in wastewater treatment as a new approach not only to purify water but also to recover metals, leveraging its selectivity properties. This dual functionality supports circular economy approaches by enabling both pollution removal and resource recovery. Due to the growing demand for critical metals, there is a need to identify new sources of these materials, for example, by using selective materials such as modified chitosan.

In addition to wastewater remediation, chitosan-based ion-imprinted sorbents could play an important role in chemical sensing and analytical chemistry, enabling the development of selective recognition elements for sensors or separation media. The incorporation of surface modifications, nanostructured additives, and hybrid materials should further expand their scope of application. Furthermore, it should be emphasized that ion-imprinting technology makes it possible to obtain materials that are selective not only between different metal ions, but also between various speciation forms of the same element. This unique property significantly broadens the potential applications of IIC sorbents, particularly in chemical analysis and speciation studies, where distinguishing between oxidation states or complex forms of a given metal is crucial. Such developments could contribute to the creation of next-generation analytical materials, providing highly selective tools for environmental monitoring, toxicological assessments, and quality control in industrial processes.

Taken together, continued progress in this field will depend on integrating sustainable synthesis strategies, computational tools, and emerging technologies such as 3D printing. If these challenges are met, IIC sorbents could evolve from laboratory curiosity into a mainstream technology for environmental protection, resource recovery, and advanced analytical applications.

## Figures and Tables

**Table 1:** Range of chitin content in various marine organisms [57,58].

Species	Chitin Content (% of Dry Weight)
Shrimp	15–40%
Crab	15–30%
Lobster	17–32%
Krill	20–30%
Octopus	5–10%
Squid	35–50%
Fungi	10–30% (cell wall)

**Table 2:** Summary of example costs for purchasing chitosan in different forms and for different applications [61].

Source	Specification	Type	Grades	Price USD/kg
Shellfish chitosan	Acid soluble	Chitosan	Food Grade	28.00
			Other Grades (Industrial, Cosmetic, Agriculture)	27.00
	Water soluble	Chitosan hydrochloride	Food, Industrial, Cosmetic, Agriculture	35.00
		Quaternary chitosan	Food, Industrial, Cosmetic, Agriculture	50.00
		Carboxymethyl chitosan	Medical Grade	188.00
		Chitosan oligosaccharide	Food Grade	78.00
			Other Grades (Industrial, Cosmetic, Agriculture)	46.00
Mushroom chitosan	Acid soluble	Chitosan	Food Grade	38.00
			Other Grades (Industrial, Cosmetic, Agriculture)	35.00
	Water soluble	Chitosan hydrochloride	Food, Industrial, Cosmetic, Agriculture	46.00
		Carboxymethyl chitosan	Medical Grade	273.00
		Chitosan oligosaccharide	Food Grade	94.00
			Other Grades (Industrial, Cosmetic, Agriculture)	55.00

**Table 3:** Summary of the main complex parameters describing the properties of heavy metal ions relevant for interpreting ion-imprinting based on [100,101,102].

Ion Metal	Atomic Weight	r (Å) Shannon (CN = 6)	CN/Geometry	Aquacomplex	Pauling Electronegativity χ	Ionic Potential z/r (Å^−1^)	M–L Bond Energy (kJ·mol^−1^)	NH_3_ (Log β)	CN^−^ (Log β)	OAc^−^ (Log β)
Cr(III)	51.99	0.62	6/octahedral	[Cr(H_2_O)_6_]^3+^	1.66	4.9	250–320	10–12	30–40	3–5
Cr(VI)	51.99	0.26	4/tetrahedral (CrO_4_^2−^)	- ^1^	1.66	13.6	500–600	-	-	-
Co(II)	58.93	0.75	6/octahedral	[Co(H_2_O)_6_]^2+^	1.88	2.68	140–180	4.7	13–20	1–3
Ni(II)	58.69	0.69	6/octahedral	[Ni(H_2_O)_6_]^2+^	1.91	2.90	160–210	8.3	16–18	1–3
Cu(II)	63.55	0.73	6/octahedral	[Cu(H_2_O)_6_]^2+^	1.90	2.74	200–260	13	27	2–4
Zn(II)	65.39	0.74	6/octahedral	[Zn(H_2_O)_6_]^2+^	1.65	2.70	150–180	8.9	10–20	1–3
Cd(II)	112.4	0.95	6/octahedral	[Cd(H_2_O)_6_]^2+^	1.69	2.11	120–150	5.4	16.9	1–2
Hg(II)	200.6	1.02	4–6/variable	[Hg(H_2_O)_6_]^2+^	2.00	1.96	250–320	19	>30	3–5
Pb(II)	207.24	1.19	8/irregular	[Pb(H_2_O)_8_]^2+^	2.33	1.68	150–190	-	10–15	2–4
As(III)	74.92	0.58	6/octahedral	-	2.18	5.2	250–320	-	-	-
As(V)	74.92	0.34	4/tetrahedral (AsO_4_^3−^)	-	2.18	14.9	450–550	-	-	-

^1^ data not determined or not applicable.

**Table 4:** Overview of chitosan sorbents imprinted with heavy metal ions.

Heavy Metal Ion as a Template	Crosslinker	Eluent	Sorption Capacity q ^1^ [mg/g]	(Relative) Selectivity Coefficient β	t_eq(s)_ [min]	pH, T [K]	C_M+_ [mg/L], Dosage [g/L]	Year Ref.
Cr(VI)	STPP	1 M NaOH 5 mM HNO_3_	41.29	-	24 h	6.7, 298	-, 0.5	2025 [110]
Cr(III)	ECH	0.1 M HCl	385.9	3.2./2.1/1.3 Co(II)/Ni(II)/Cd(II)	120	3, 298	8000, 2	2024 [111]
Cr(III)	Glyoxal	EDTA	250.0	15.2/13.3/11.0 Fe(III)/Cu(II)/Eu(III)	30	5, 303	400, 1.0	2022 [112]
Cr(VI)	ECH	0.2 M HNO_3_	550.5	7.35	30	3, 313	500, 0.5	2017 [113]
Co(II)	ECH	1% HCl	-	-	60	-, 313	100, 10	2022 [118]
Co(II)	ECH	0.5 M HNO_3_	100.0	42/11/2 Ni(II)/Cd(II)/Pb(II)	10	8, -	40, 0.2	2016 [119]
Co(II)	ECH	1 M H_2_SO_4_	92.2 µmol/g	2.81	3 days	4.8, -	4 mM, -	2012 [89]
Co(II)	KH-560	1 M H_2_SO_4_	31.5	8.23	90	4.0, 293	400, 4	2011 [120]
Co(II)	KH-560	3 M HNO_3_	22.5	13.8/11.5/9.4 Ni(II)/Pb(II)/Sr(II)	300	6.0, 298	500, 4	2010 [121]
Ni(II)	ECH STTP	5% Na-EDTA	18.5	5.6/8.4 Cu(II)/Zn(II)	60	7.0, 298	25, 0.5	2021 [125]
Ni(II)	GLA	0.1 M HCl	109.9	5.45	40	5.0, 293	100, 0.2	2021 [126]
Ni(II)	ECH	1 M HCl	29.5	56.9/55.2/49.2 Co(II)/Ca(II)/Mn(II)	240	4.0, 298	100, 50	2020 [127]
Ni(II)	Glyoxal	0.05 M EDTA	135.0	12.1/11.3/10.2 Co(II)/Cd(II)/Pb(II)	120	5.0, 303	400, 1.0	2020 [128]
Ni(II)	ECH	0.007 M EDTA	0.99 mmol/g	2.4	24 h	7.0, 298	2 mmol/L, 3.0	2019 [129]
Ni(II)	GLA	0.1 M HCl	82.8	3.53	300	6.0, 323	400, 1.0	2018 [130]
Ni(II)	MBA	0.1 M EDTA	51.6	7.33	10	6.0, 303	60, -	2018 [131]
Ni(II)	ECH	1 M H_2_SO_4_	69.9	2.6/10.5 Co(II)/Mn(II)	120	6.0, 313	2000, 1.0	2017 [132]
Ni(II)	STPP ECH	5% EDTA	-	2.3/6.0/2.2 Cd(II)/Cu(II)/Pb(II)	10	5.0–7.0, 293	-, 0.25	2016 [133]
Ni(II)	-	10 g/L EDTA	500	1.2/2.0/2.1 Cu(II)/Ag(I)/Zn(II)	360	5.0–6.0, 298	8000, 2.5	2015 [134]
Ni(II)	KH-560	2 M HNO_3_	33.2	4.8/6.5/28.1 Co(II)/Hg(II)/Mn(II)	-	5.0, -	400, 16	2010 [135]
Cu(II)	ECH	1 M H_2_SO_4_	85.1	3.3/2.7/2.4 Zn(II)/Ni(II)/Co(II)	180	5.0, 298	900, 20	2021 [140]
Cu(II)	GLA	0.05 EDTA	82.6	11.3/4.6/1.9 Cr(III)/Ni(II)/Fe(III)	150	4.5, 298	1000, 3.5	2020 [144]
Cu(II)	ECH	0.1 M HCl	143	9.2/8.5/8.8 Co(II)/Ni(II)/Pb(II)	-	5.9, 303	400, 1.0	2020 [148]
Cu(II)	-	0.1 M H_2_SO_4_	119.4	-	250	5.0, 298	500, 1.0	2020 [152]
Cu(II)	GLA	1 M HCl	83.3	2.3 Zn(II)	8 h	5.7, 298	60, 0.2	2019 [153]
Cu(II)	BADGE	1 M HCl	17.5	1.5/7.9/60.6 Zn(II)/Pb(II)/Cd(II)	120	5.0, -	200, 0.4	2019 [151]
Cu(II)	GLA	0.1 M AcO	163.1	18.2/69.4/85.1 Ni(II)/Mn(II)/Mg(II)	60	6.0, 318	500, 1.0	2019 [149]
Cu(II)	GLA	0.05 M EDTA	259.56	7.0/15.2/64.5 Zn(II)/Ni(II)/Pb(II)	30	6.0/298	500, 1.0	2018 [155]
Cu(II)	GLA	AcO	261.3	24.1/30.2/32.4 Pb(II)/Ni(II)/Zn(II)	100	7.0, 298	100, 1.0	2019 [145]
Cu(II)	GLA	0.2 M HCl	142.9	2.1/2.4/4.2 Zn(II)/Cd(II)/Co(II)	120	6.0, 293	500, 1.0	2017 [156]
Cu(II)	GLA	0.05 M EDTA	82.0	2.1/2.2/2.2 Co(II)/Mn(II)/Pb(II)	6000	5.5, 303	400, -	2017 [146]
Cu(II)	ECH	3 M HCl	4.8 mmol/L	52.3/74.7/105 Ni(II)/Cd(II)/Co(II)	-	5.7, 303	1 mol, 3.3	2016 [150]
Cu(II)	KH-560	1 M HCl	31.4	6.9/7.7 Ni(II)/Zn(II)	30	5.0, 298	600, 5.0	2015 [141]
Cu(II)	GLA	0.5 M HCl	232.6	-	-	5.5, 293	150, -	2015 [154]
Cu(II)	GLA	1 mg/L HCl	109.9	4.0/10.6/14.4 Ni(II)/Cd(II)/Zn(II)	180	5.0, 298	900, 20	2015 [142]
Cu(II)	ECH	1% HCl	201.7	4.0/4.8 Zn(II)/Pb(II)	300	5.0, 298	500, 1.0	2012 [147]
Cu(II)	ECH STPP	0.2 M EDTA	46.3	2.3/2.7 Zn(II)/Ni(II)	6 h	5.5, 298	300, 0.5	2008 [143]
Zn(II)	EGDMA TEOS	1 N HCl	3.1	-	-	7.0, -	30, 2.0	2020 [161]
Zn(II)	EDGMA	0.005 M EDTA	71.4	-	150	8.5, 298	500, 0.2	2017 [162]
As(V)	GLA	0.1 M HCl	625	11.2/6.2/5.3 Ca(II)/Na(I)/K(I)	20 s	4.0, 303	200, 0.7	2023 [168]
As(III)	EGDMA	0.5 M HCl	37.0	29.4	48	7.25, 303	300, 0.2	2020 [169]
As(III)	GLA	0.5 M HCl	4.1	7.4/10.0/17.8 Cd(II)/Zn(II)/Mg(II)	10 h	6.0, 303	100, 4.0	2011 [170]
As(III)								2011 [171]
As(III)	GLA	0.5 M HCl	9.4	502.3/12.6/12.3 Fe(III)/Pb(II)/Cd(II)	6 h	5.0, 303	100, 5.0	2011 [172]
Cd(II)	STPP	0.01 M H_2_SO_4_ 0.2 M NaCl	1.05 mmol/g	2.0/2.0 Ni(II)/Co(II)	48 h	6.5, -	8 mM, 0.65	2025 [177]
Cd(II)	-	0.06 M Na-EDTA	535	3.9/3.1/3.2 Ni(II)/Pb(II)/Mn(II)	4 h	-,	800, 0.5	2024 [179]
Cd(II)	GLA	10% HCl in ethanol	45.87	4.6/3.0/2.8 Pb(II)/Mg(II)/Zn(II)	30	6, 298	400, 1.0	2023 [180]
Cd(II)	ECH	0.1 M EDTA	305	23.2/21.3/20.1 Pb(II)/Cu(II)/Ni(II)	3 h	6, 303	400, 1.0	2023 [182]
Cd(II)	STPP	0.01 M H_2_SO_4_ 0.2 M NaCl	1.05 mmol/g	-	48 h	7.5, 298	1500, 0.65	2022 [178]
Cd(II)	GLA	0.1 M AcO	70.5	12.9/3.1/1.1 Pb(II)/Fe(III)/Zn(II)	4 h	5, 298	75, 1.0	2021 [184]
Cd(II)	GLA	1 M HNO_3_	26.1	3.9/3.3/2.1 Cu(II)/Cr(II)/Pb(II)	60	6, 298	500, 0.5	2019 [181]
Cd(II)	ECH	1.2 M HCl	167	3.4/3.3/3.2 Ag(I)/Cu(II)/Zn(II)	120	6, 303	5 mmol, 1.25	2018 [183]
Cd(II)	ECH	1.2 M HCl	38.6	3.0/2.5/2.5 Ni(II)/Cu(II)/Zn(II)	90	-, 293	50, 2.5	2017 [185]
Hg(II)	STPP	0.5 M EDTA	-	-	-	-	-	2024 [188]
Hg(II)	GLA	0.1 M EDTA	315	17.9/16.1/15.4 Pb(II)/Cd(II)/Cu(II)	70	5, 303	500, 1.0	2022 [189]
Pb(II)	GLA	0.1 M HCl	82.0	25.8/28.9/28.5 Cu(II)/Ca(II)/Mg(II)	25 s	5, 303	200, 0.7	2023 [168]
Pb(II)	ECH	1 M H_2_SO_4_	82.0	13.4/9.1/7.1 Co(II)/Ni(II)/Cd(II)	90	4, 303	500, 5	2023 [192]
Pb(II)	ECH	0.1 M EDTA	124.1	3.9/3.4/3.0 Cu(II)/Cd(II)/Ni(II)	120	6, 298	400, 2.5	2020 [193]
Pb(II)	MBA	0.2 M HNO3	30.1	17.3/16.0/15.5 Ni(II)/Cu(II)/Co(II)	15	7, 313	100, 0.7	2020 [194]
Pb(II)	GLA	0.1 M EDTA	83.2	59.5/24.8/20.5 Cd(II)/Zn(II)/Ni(II)	80	6, 308	100, 1.0	2020 [195]
Pb(II)	BADGE	0.2 M Na-EDTA	0.7	5.06/0.06 Cu(II)/Zn(II)	-	-	500, 1.0	2023 [197]
Pb(II)	GLA	2 M HCl	76.6	-	-	4, 298	500, -	2019 [202]
Pb(II)	ECH	EDTA	116.3	7.9/4.7/3.6 Ni(II)/Cd(II)/Cu(II)	480	5, -	800, 2.5	2019 [199]
Pb(II)	ECH	EDTA	69.5	2.3/2.2/2.1 Cu(II)/Cd(II)/Ni(II)	-	5, -	200, 2.5	2017 [200]
Pb(II)	PEGDE	Na-EDTA	40 mmol/g	-	60	5, -	-, 1.0	2017 [198]
Pb(II)	BADGE	1 M HCl	167.1	-/-/- Ni(II)/Cd(II)/Cu(II)	250	5, -	500, 0.6	2014 [196]
Pb(II)	ECH	HCl	74.1	-	240	5, 298	10,0.1	2011 [88]
Pb(II)	ECH	0.05% EDTA	139.6	-	180	5–7, 298	1000, 1.0	2010 [201]

^1^ The maximum sorption capacity, calculated using the Langmuir model or another model, depends on the data from the article.

## Data Availability

Not applicable.

## Supplementary Materials

The following supporting information can be downloaded at: https://www.mdpi.com/article/10.3390/ijms27073183/s1.

## Abbreviations

The following abbreviations are used in this manuscript:

AIBN	2-Azobisisobutyronitrile
BADGE	Bisphenol A Diglycidyl Ether
CMC	Carboxymethyl Chitosan
CNT	Carbon Nanotube
CP	Controlled Polymer
DDA	Degree of Deacetylation
DNA	Deoxyribonucleic Acid
ECH	Epichlorohydrin
EDC	1-Ethyl-3-(3-dimethylaminopropyl)carbodiimide
EDTA	Ethylenediaminetetraacetic Acid
EDTA-Na	Sodium Salt of Ethylenediaminetetraacetic Acid
EGDMA	Ethylene Glycol Dimethacrylate
FAAS	Flame Atomic Absorption Spectroscopy
GLA	Glutaraldehyde
GO	Graphene Oxide
4-HBA	4-Hydroxybenzoic Acid
IIC	Ion-Imprinted Chitosan
IIP	Ion-Imprinted Polymer
IP	Imprinted Polymer
k’	Relative Selectivity Coefficient
K_d_	Partition Constant
KH-560	(3-Glycidyloxypropyl)trimethoxysilane
MBA	N,N’-Methylenebisacrylamide
MIC	Molecular-Imprinted Chitosan
MIP	Molecular-Imprinted Polymer
NHS	N-Hydroxysuccinimide
NIP	Non-Imprinted Polymer
PAN	1-(2-Pyridylazo)-2-naphthol
PEGDE	Poly(ethylene)-glycol Diglycidyl Ether
PVA	Polyvinyl Alcohol
q	Sorption Capacity (mg/g)
R%	Recovery Percentage/Removal Percentage (%)
SEM	Scanning Electron Microscopy
STPP	Sodium Tripolyphosphate
TEOS	Tetraethyl Orthosilicate
UA-µ-SPE	Ultrasonic-Assisted Solid-Phase Microextraction
